# The shifting lipidomic landscape of blood monocytes and neutrophils during pneumonia

**DOI:** 10.1172/jci.insight.164400

**Published:** 2024-02-22

**Authors:** Alex R. Schuurman, Osoul Chouchane, Joe M. Butler, Hessel Peters-Sengers, Sebastiaan Joosten, Xanthe Brands, Bastiaan W. Haak, Natasja A. Otto, Fabrice Uhel, Augustijn Klarenbeek, Christine C.A. van Linge, Antoine van Kampen, Mia Pras-Raves, Michel van Weeghel, Marco van Eijk, Maria J. Ferraz, Daniël R. Faber, Alex de Vos, Brendon P. Scicluna, Frédéric M. Vaz, W. Joost Wiersinga, Tom van der Poll

**Affiliations:** 1 Center for Experimental and Molecular Medicine (CEMM), Amsterdam University Medical Centers - Location AMC, University of Amsterdam, Amsterdam, Netherlands.; 2 Université Paris Cité, INSERM UMR-S1151, CNRS UMR-S8253, Institut Necker-Enfants Malades, Paris, France.; 3 Médecine Intensive Réanimation, AP-HP, Hôpital Louis Mourier, DMU ESPRIT, Colombes, France.; 4 Department of Clinical Epidemiology, Biostatistics and Bioinformatics, Amsterdam University Medical Centers - Location AMC, University of Amsterdam, Amsterdam, Netherlands.; 5 Core Facility Metabolomics, Amsterdam UMC, Amsterdam, Netherlands.; 6 Department of Clinical Chemistry and Pediatrics, Laboratory Genetic Metabolic Diseases, Emma Children’s Hospital, Amsterdam University Medical Centers - Location AMC, University of Amsterdam, Amsterdam, Netherlands.; 7 Amsterdam Gastroenterology Endocrinology Metabolism, Inborn Errors of Metabolism, Amsterdam, Netherlands.; 8 Leiden Institute of Chemistry, University of Leiden, Netherlands.; 9 Department of Internal Medicine, BovenIJ Hospital, Amsterdam, Netherlands.; 10 Department of Applied Biomedical Science, Faculty of Health Sciences, Mater Dei Hospital, and; 11 Center for Molecular Medicine and Biobanking, University of Malta, Msida, Malta.; 12 Division of Infectious Diseases, Amsterdam University Medical Centers - Location AMC, University of Amsterdam, Amsterdam, Netherlands.

**Keywords:** Immunology, Infectious disease, Innate immunity, Molecular biology, Neutrophils

## Abstract

The lipidome of immune cells during infection has remained unexplored, although evidence of the importance of lipids in the context of immunity is mounting. In this study, we performed untargeted lipidomic analysis of blood monocytes and neutrophils from patients hospitalized for pneumonia and age- and sex-matched noninfectious control volunteers. We annotated 521 and 706 lipids in monocytes and neutrophils, respectively, which were normalized to an extensive set of internal standards per lipid class. The cellular lipidomes were profoundly altered in patients, with both common and distinct changes between the cell types. Changes involved every level of the cellular lipidome: differential lipid species, class-wide shifts, and altered saturation patterns. Overall, differential lipids were mainly less abundant in monocytes and more abundant in neutrophils from patients. One month after hospital admission, lipidomic changes were fully resolved in monocytes and partially in neutrophils. Integration of lipidomic and concurrently collected transcriptomic data highlighted altered sphingolipid metabolism in both cell types. Inhibition of ceramide and sphingosine-1-phosphate synthesis in healthy monocytes and neutrophils resulted in blunted cytokine responses upon stimulation with lipopolysaccharide. These data reveal major lipidomic remodeling in immune cells during infection, and link the cellular lipidome to immune functionality.

## Abstract 2

<p>Untargeted lipidomics of human monocytes and neutrophils unveils major lipidomic remodeling during pneumonia&nbsp;and links the cellular lipidome to immune functionality.</p>

## Graphical abstract

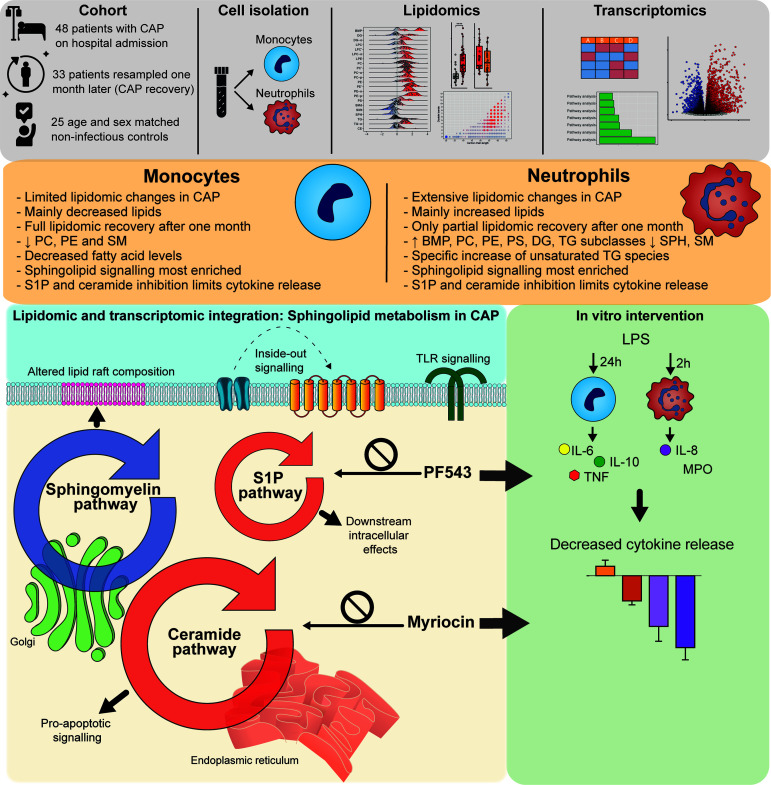

## Introduction

Through decades of research, a multitude of pathophysiological mechanisms that shape the host response during infection has been identified (1, 2). Herein, monocytes and neutrophils have emerged as key innate immune cell subsets. Monocytes orchestrate the immune response by exerting both proinflammatory and inflammation-resolving functions (3). Neutrophils are potent antimicrobial effector cells — by way of phagocytosis, degranulation of toxic factors, and formation of neutrophil extracellular traps (NETs) — that can also shape the local milieu through cytokine and chemokine production (4).

Our current understanding of the immune cell response during infection mainly derives from studies that focused on a certain set of cell state indicators, such as cell-surface marker proteins, cytokine production capacity, or the transcriptomic profile. The lipidome of immune cells during infection has remained uncharted thus far, despite its putative importance. Lipids are a prerequisite for the existence of cells and essential for their structural and bioactive functions (5). By forming a bilayer, lipids are the building blocks of all membranes, providing structure and compartmentalization. The cell membrane composition can reflect intra- and extracellular perturbations, but also actively facilitate signal transduction, cell adhesion, and endo/exocytosis (6). As bioactive molecules, lipids play a key role in many cellular processes such as energy metabolism, inflammatory responses, and cell-cell signaling (7).

Evidence of an important role for lipids during infection is mounting. Several studies reported lipid signatures in plasma of patients with sepsis that correlated with therapy responsiveness and mortality (8–10). Functionally, it was demonstrated that macrophage phenotypes — ranging from pro- to antiinflammatory — can be actively altered through modulation of fatty acid β-oxidation (11). The lipidomes of monocytes and neutrophils were recently explored in healthy volunteers, revealing changes in their composition upon in vitro short-term stimulation with a calcium ionophore (12).

In this study, we performed untargeted lipidomic analysis of blood monocytes and neutrophils from patients with community-acquired pneumonia (CAP) on hospital admission — and 1 month thereafter — and matched control volunteers. We describe a profound impact of pneumonia on the lipidome of blood monocytes and neutrophils, with both common and distinct changes between these cell types. Integration with cell transcriptomes highlighted altered sphingolipid metabolism pathways, and ex vivo inhibition of ceramide and sphingosine-1-phosphate (S1P) synthesis resulted in a reduced cytokine production capacity upon exposure to lipopolysaccharide (LPS). Together, these data delineate a shifting immune cell lipidomic landscape during infection, and link the cellular lipidome to immune functionality.

## Results

### Patients and controls.

**Figure 1:**
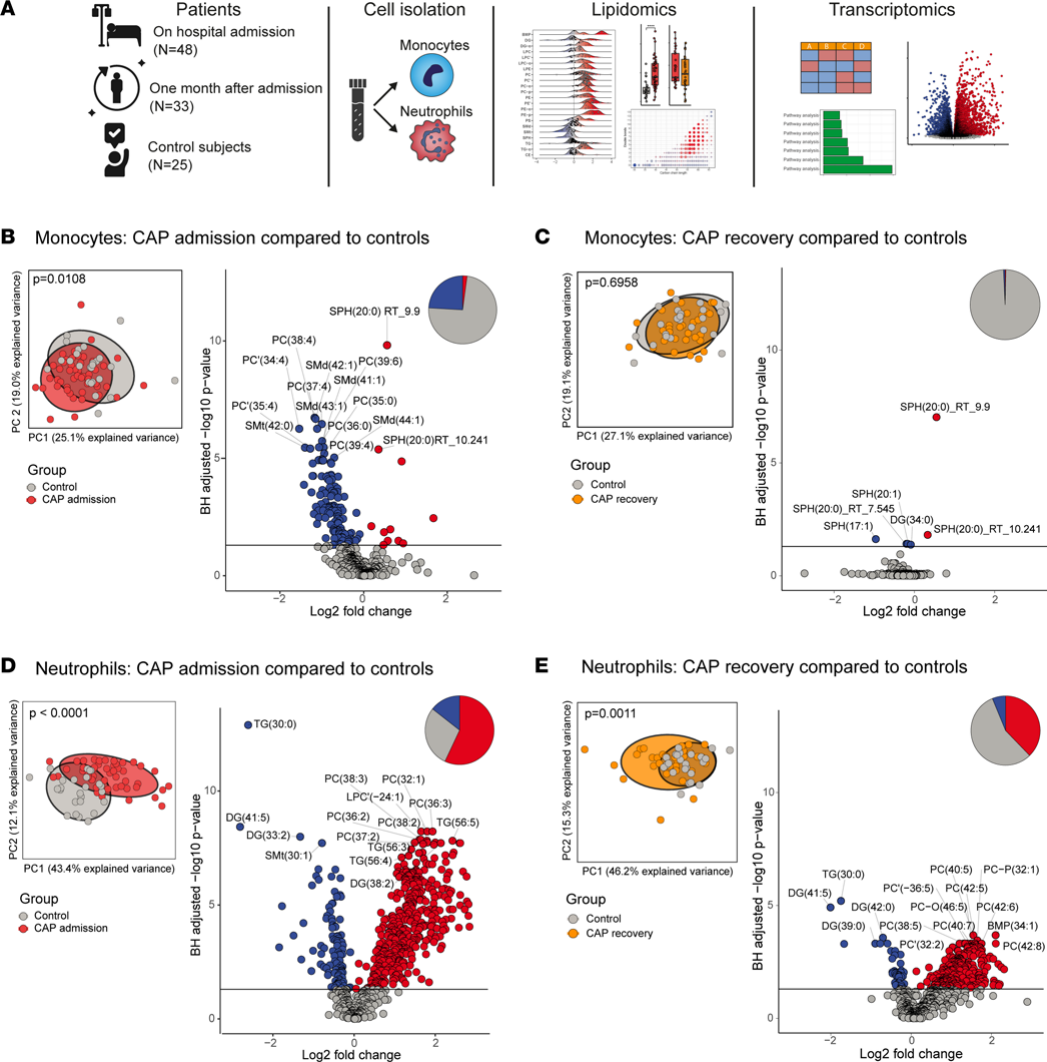
The lipidomes of monocytes and neutrophils are distinctly altered during pneumonia. (**A**) Study overview. (**B**) Principal Component Analysis (PCA) and volcano plot comparing the monocyte lipidome between patients with CAP and matched noninfectious controls. In the PCA plot, each dot represents a participant and the color represents the group. The *x* and *y* axes show the first and PC with the percentage of explained variance, respectively. Difference between groups on the PCA plot was tested by Wilcoxon’s rank-sum test of the first PC coordinates. The volcano plot shows the differential abundance of lipids between patients with CAP and controls. Each dot represents a lipid species, while the color indicates whether the lipid is significantly more abundant (red), significantly less abundant (blue), or not significantly altered (gray). The *x* axis denotes the log_2_(fold change) between groups, while the *y* axis shows the Benjamini-Hochberg–adjusted –log_10_(*P* value). The pie chart represents the whole lipidome and gives an indication of what percentage of lipids is more abundant, less abundant, or unchanged. (**C**) Identical to panel **A**, but here for CAP recovery monocytes versus control monocytes. (**D**) Identical to panel **B**, but here for neutrophils. (**E**) Identical to panel **C**, but here for neutrophils.

We isolated and profiled blood monocytes and neutrophils from 48 patients hospitalized due to CAP (CAP admission) and 25 noninfectious volunteers (controls) visiting the outpatient clinic for a blood draw, without signs of acute infection (Table 1 and Figure 1A). Samples from patients were obtained within 24 hours of admission to the general hospital ward. One month after hospital admission, we resampled 33 of the patients with CAP (CAP recovery). Patients with CAP and controls were matched for age and sex, while comorbidities and chronic medications largely overlapped between groups. Patients were moderately ill based on disease severity scores, which was also reflected in a mean hospital length of stay of 6 days, a low number of intensive care unit admissions, and zero mortality within 1 month after admission.

### Lipidomic measurements.

Lipidomic analysis of isolated monocytes and neutrophils by high-performance liquid chromatography coupled to mass spectrometry (HPLC-MS) yielded a primary data set of 521 monocyte and 706 neutrophil lipid species, which were normalized to an extensive set of internal standards as previously reported (13, 14) (see Methods for a full description). We also annotated a second data set of lipid species that lacked a specific internal standard, and these were normalized to the phosphatidylethanolamine (PE) internal standard. We only analyzed these secondary data in support of the primary data set (with internal standards), and only on a lipid class–wide level.

### The lipidomes of monocytes and neutrophils are distinctly altered during pneumonia.

We first explored the impact of CAP on the lipidome of blood monocytes and neutrophils in an unsupervised manner. Principal Component Analysis (PCA), an analysis tool based on dimensionality reduction (15), showed a moderate but significant difference between the monocyte lipidomes of patients and controls (Figure 1B, Wilcoxon’s test on PC1, *P* = 0.0108). To investigate these differences on a lipid-specific level, we visualized monocyte lipidomic differences in a volcano plot (Figure 1B). One-quarter of the monocyte lipidome was significantly altered in CAP after correction for multiple testing (correcting for all 522 monocyte lipids), mainly comprising lipids that were decreased in patients (24% down, 2% up, 74% unchanged). The most significantly decreased lipids mainly included sphingomyelin (SM) and phosphatidylcholine (PC) species. The comparison between CAP recovery samples and controls yielded considerably fewer differences, with no significant difference on the PCA plot and fewer differentially abundant lipids: <1% down, <1% up, and 99% unchanged, indicating that CAP-induced lipidomic changes were transient in monocytes (Figure 1C). We took a similar approach in analyzing neutrophils, and found that, relative to monocytes, the neutrophil lipidome appeared more divergent between patients and controls (Figure 1D, *P* < 0.0001). Indeed, strikingly, the majority of the neutrophil lipidome was significantly different between patients with CAP and controls, mainly constituting lipids with increased abundance in patients’ neutrophils (14% down, 57% up, 29% unchanged). The top 15 differential lipids were all more abundant during CAP and mostly composed of bis(monoacylglycero)phosphate (BMP), diacylglycerol (DG), and PC species. Notably, 1 month after hospital admission a substantial part of the neutrophil lipidome remained different as compared with noninfectious controls (Figure 1E; 6% down, 38% up, and 56% unchanged), possibly reflecting persistent changes in myelopoiesis. An overview of the fold change and significance of all annotated lipid species in these 4 comparisons is provided in Supplemental Table 1; supplemental material available online with this article; https://doi.org/10.1172/jci.insight.164400DS1 In summary, we found profound and distinct lipidomic changes in monocytes and neutrophils during pneumonia. Changes in the monocyte lipidome were modest and transient, while 1 month after hospital admission the neutrophil lipidome remained partly different from controls.

### Class-wide lipidomic changes in monocytes and neutrophils of patients with CAP.

**Figure 2:**
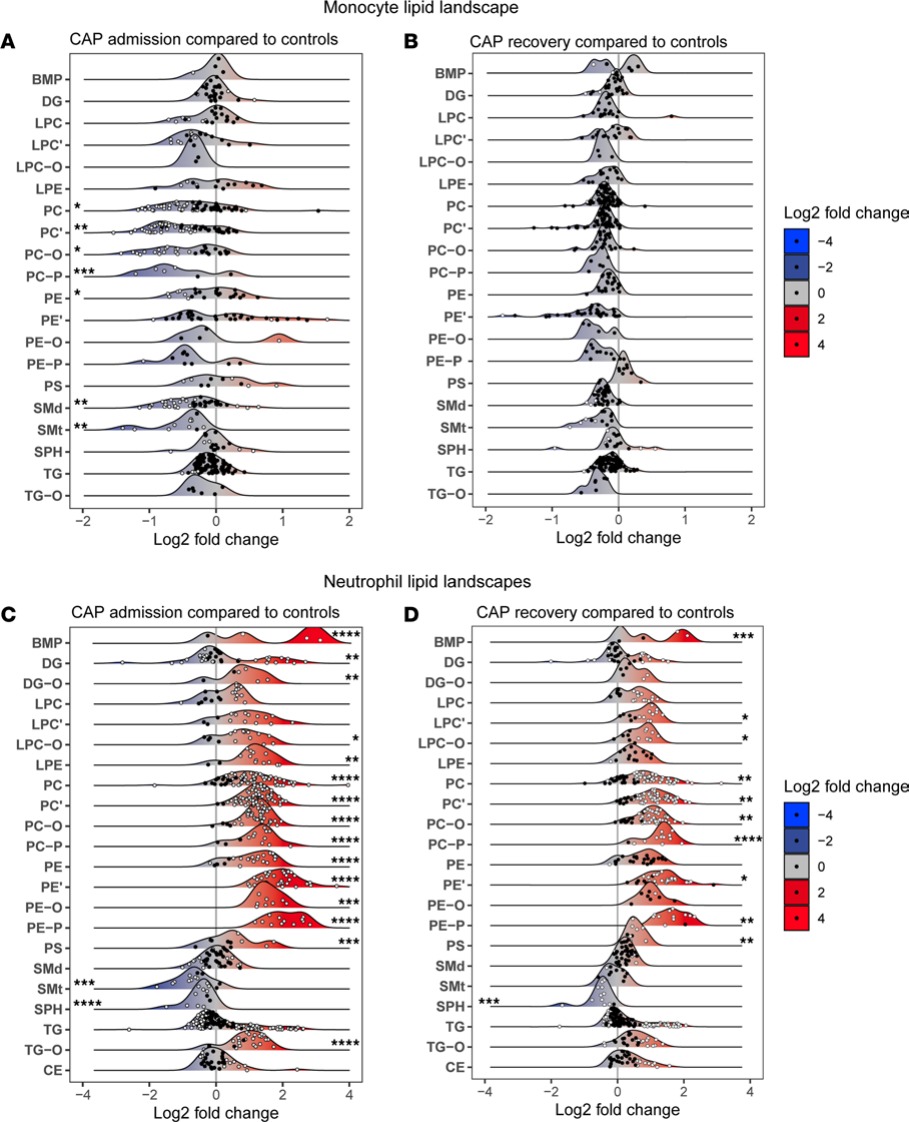
The lipid landscapes of monocytes and neutrophils during pneumonia. (**A**) Monocyte lipid landscape plot, comparing patients with CAP at admission to controls. Each dot represents a lipid species, which are grouped per lipid class. The color of the dots indicates whether the lipid was significantly different (white) between the groups after Benjamini-Hochberg correction for all annotated lipids. The *x* axis shows the log_2_(fold change) between groups for each lipid. The *y* axis indicates the different lipid classes, with a ridge plot per lipid class that shows the distribution of the lipids within their respective classes. On the edges of the plot, stars denote class-wide significant differences as determined by Wilcoxon’s rank-sum test of aggregated values per class (a sum of all lipids per class per participant). All box-and-whisker plots of this analysis are shown in Supplemental Figure 1A. Stars on the left of the plot indicate a significant class-wide decrease, and stars on the right of the plot indicate a significant class-wide increase. A *P* value below 0.05 was considered significant. *P < 0.05, **P < 0.01, ***P < 0.001, ****P < 0.0001. (**B**) Identical to panel **A**, but here comparing CAP recovery monocytes to control monocytes. All box-and-whisker plots of this analysis are shown in Supplemental Figure 1B. (**C**) Identical to panel **A**, but here for neutrophils. All box-and-whisker plots of this analysis are shown in Supplemental Figure 2A. (**D**) Identical to panel **B**, but here for neutrophils. All box-and-whisker plots of this analysis are shown in Supplemental Figure 2B.

As uniform changes in groups of related lipids may give more robust information on certain biological processes than differential single lipids, we grouped all lipids based on their respective biochemical classes (Supplemental Table 2). Per cell type, we compared both patients with CAP at hospital admission to controls and CAP recovery samples to controls. Class-wide differences were calculated by aggregating all lipid abundances per class per individual. All class-wide comparisons are shown in box-and-whisker plots, depicted in Supplemental Figures 1 and 2. To illustrate the direction, distribution, and significance of change for each lipid within a class consisting of at least 4 species, we constructed a lipid landscape plot for each comparison (Figure 2).

Monocytes from patients with CAP had a lower relative abundance of several lipid classes when compared with controls (Figure 2A and Supplemental Figure 1A). We found a significant decrease in the 2 major cell membrane constituents: the glycerophospholipid classes PC and PE, which have a structural role but also regulate membrane fluidity, protein interactions, and membrane fusion (16). All PC subclasses were decreased in monocytes from patients with CAP, including alkyl-ether (O) and alkenyl-ether plasmalogen lipids (P). The 2 SM classes (SMd and SMt) were also significantly less abundant in monocytes of patients with CAP. In line with our findings (displayed in Figure 1), a paired comparison of CAP admission and CAP recovery samples showed a strong trend of reversal of the observed lipidomic changes (Supplemental Figure 3A). Importantly, monocytes obtained after CAP recovery showed no class-wide differences anymore when compared to controls (Figure 2B and Supplemental Figure 1B), indicating that most lipidomic changes in monocytes normalize within 1 month after hospital admission.

Next, we used the same approach in analyzing neutrophils, which revealed a markedly altered lipidome in patients with CAP when compared with controls (Figure 2C and Supplemental Figure 2A). We found a highly significant increase in the BMP class in patients’ relative to controls’ neutrophils. The increase in BMP lipids could point to increased lysosomal and phagosomal content and activity in preparation for neutrophil effector functions, as BMPs are exclusively present in late endosomes and lysosomes (17). Neutrophil DG, the precursor of triacylglycerol (TG), showed a class-wide increase in CAP when compared with controls, although DG and TG classes also comprised species that were significantly decreased. DGs can be phosphorylated by diacylglycerol kinases to produce the lipid mediator phosphatidic acid (PA), which was also highly increased in CAP patients (Supplemental Figure 2A) (18). The lysophospholipid classes lysophosphatidylcholine (LPE) and lysophosphatidylethanolamine (LPE), mainly generated by phospholipase A2–mediated (PLA2-mediated) hydrolysis of PC/PE, both comprised many increased species, although only the LPE class was increased as a whole. In sharp contrast with monocytes, we noted a highly significant increase in PC and PE in neutrophils during CAP — including the ether lipids (with an alkyl [-O] or alkenyl ether [-P] bond) — suggesting major membrane remodeling. Ether lipids are synthesized in the peroxisome, and peroxisomal ether lipid production was found to be crucial for neutrophil membrane composition and viability during inflammation (19). Sphingolipid classes SMt and SPH, the latter being the backbone of sphingolipids, were significantly decreased in neutrophils from patients with CAP when compared with controls. The comparison of CAP recovery and CAP admission samples indicated reversal of several changes, although this was mainly significant for PC and PE (Supplemental Figure 3B). In line with the observed persistent changes in Figure 1, several lipid classes in neutrophils obtained after CAP recovery still differed from control neutrophils, indicating only partial lipidomic recovery 1 month after hospital admission for pneumonia (Figure 2D).

Taken together, our results demonstrate both shared and distinct class-wide lipidomic changes in monocytes and neutrophils of patients with CAP when compared with controls. SM classes were significantly less abundant in both monocytes and neutrophils from patients with CAP, possibly revealing a common change in these cell types during pneumonia.

### Proportional changes in monocyte and neutrophil subsets between groups.

To explore why monocyte lipidomes normalized after 1 month, while neutrophil lipidomes were persistently altered, we analyzed the proportional differences of monocyte and neutrophil subsets between the groups. First, we identified classical (CD14^+^CD16^−^), intermediate (CD14^+^CD16^+^), and nonclassical (CD14^dim^CD16^+^) monocyte subsets. Patients with CAP at hospital admission had a significantly higher proportion of intermediate monocytes, with relatively fewer classical and nonclassical monocytes (Supplemental Figure 3C). Interestingly, monocyte subset proportions were again equally distributed between CAP recovery and healthy control samples, which may help explain why the monocyte lipidome was fully normalized 1 month after hospital admission. Next, we analyzed neutrophil subsets. It is well documented that, in a process called emergency myelopoiesis, immature neutrophils can arise from the bone marrow and emerge in the peripheral blood in response to infection (20). We indeed identified the presence of some immature (CD16^lo/–^) neutrophils in the neutrophil fraction of patients with CAP, but proportions were not significantly different between groups (Supplemental Figure 3D). To further explore the concept of emergency myelopoiesis in our cohort, we also analyzed the polymorphonuclear cell (PBCM) fraction, from which the majority of immature neutrophils are normally retrieved due to their lower cellular density. We found a striking increase in both mature and immature low-density granulocytes in patients with CAP at hospital admission, and to a lesser extent at CAP recovery (Supplemental Figure 3E, see Supplemental Figure 3F for the gating strategy), hinting at a partially maintained state of emergency myelopoiesis. Together, the flow cytometry analyses suggest that shifting proportions of cellular subsets and/or states may underpin part of the observed lipidomic differences between the groups, although more work is needed — for instance through single-cell lipidomics (21) — to establish direct links.

### Elevated triglycerides in neutrophils from CAP patients are polyunsaturated.

Neutrophils from CAP patients showed a bimodal distribution of TG, DG, and (in the recovery phase only) PC lipids, i.e., some lipids within these classes were significantly decreased in patients with CAP relative to controls, while others were significantly increased (Figure 2C). To further investigate these classes in both cell types, we assessed whether the degree of unsaturation of lipids was linked to the disease state. Structurally, TG, DG, and PC lipids contain fatty acids — 3 in TGs, 2 in DGs, and variable in PCs — which can be either saturated, monounsaturated, or polyunsaturated (PUFA) (5). The number of double bonds in their chemical structure indicates the degree of unsaturation of the fatty acids. As such, a TG with more than 4 double bonds consists by definition of at least 1 polyunsaturated fatty acid.

**Figure 3:**
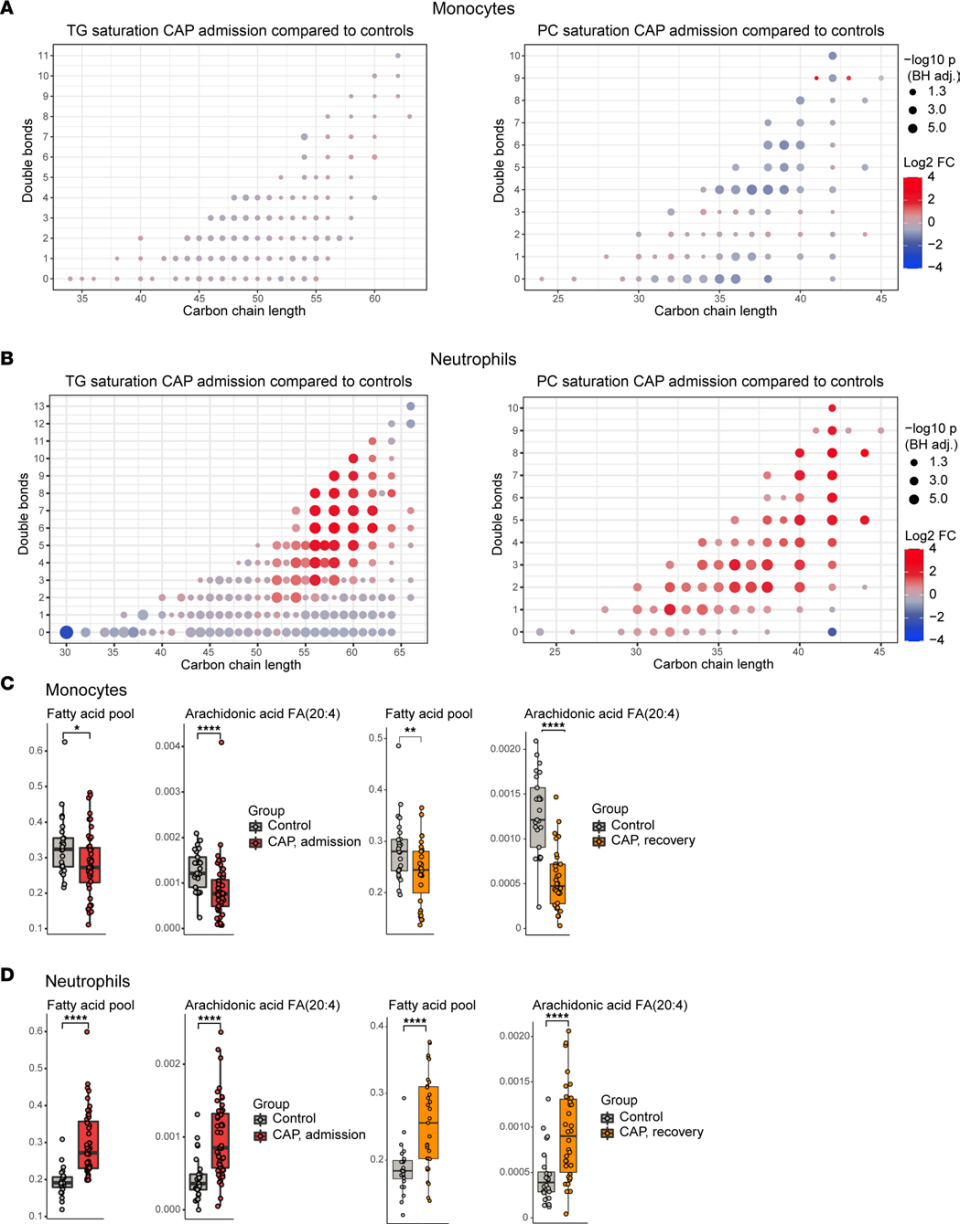
Lipid saturation and fatty acids in monocytes and neutrophils during CAP. (**A**) Dot plots of triacylglycerol and phosphatidylcholine saturation in monocytes during CAP. Each dot represents a lipid species. The color of the dots indicates the log_2_(fold change) between the groups, and the size of the dot is proportional to the Benjamini-Hochberg–adjusted –log_10_(*P* value) of this change. The *x* axis indicates the cumulative carbon chain length, and the *y* axis shows the total amount of double bonds. (**B**) Identical to panel **A**, but here for neutrophils. (**C**) Box-and-whisker plots comparing the total free fatty acid pool (the sum of all free fatty acid species) and arachidonic acid in monocytes between patients with CAP and controls, and the CAP patients after recovery to controls. Significance was determined by Wilcoxon’s rank-sum test. A *P* value below 0.05 was considered significant. *P < 0.05, **P < 0.01, ***P < 0.001, ****P < 0.0001. (**D**) Identical to panel **C**, but here for neutrophils.

The saturation of monocyte TG and PC did not show a clear pattern (Figure 3A). In neutrophils, however, specifically unsaturated TGs were significantly more abundant in patients with CAP, while saturated TG species were decreased (Figure 3B). Of note, comparison of neutrophil TG species in CAP recovery to those in CAP admission showed a reverse image (Supplemental Figure 4A). We detected similar changes in DGs, although differences were less clear due to a lower number of lipids in this class (Supplemental Figure 4B). In neutrophil PCs, all species were uniformly increased during CAP, irrespective of the degree of saturation (Figure 3B). As altered fatty acid metabolism might underpin the enrichment of TGs with PUFAs, we analyzed fatty acids in both cell types as part of the lipidomics experiment. The pool of annotated fatty acids — mostly comprising saturated and monosaturated lipids — was clearly decreased in monocytes and increased in neutrophils during CAP (Figure 3, C and D). Of note, these changes did not normalize 1 month after hospital admission. The only annotated PUFA was arachidonic acid, a well-known eicosanoid precursor, which showed a distribution similar to the whole fatty acid pool in both cell types (Figure 3, C and D). Collectively, these findings indicate that neutrophils, and not monocytes, in patients with CAP are enriched with PUFA-containing TGs.

### Transcriptomic analysis highlights altered sphingolipid metabolism in both monocytes and neutrophils of patients with CAP.

To obtain further insight into the meaning and direction of the observed lipidomic changes, we performed RNA sequencing in aliquots of the same monocytes (45 CAP admission, 23 healthy control, 31 CAP recovery) and neutrophils (35 CAP admission, 13 healthy control, 17 CAP recovery).

**Figure 4:**
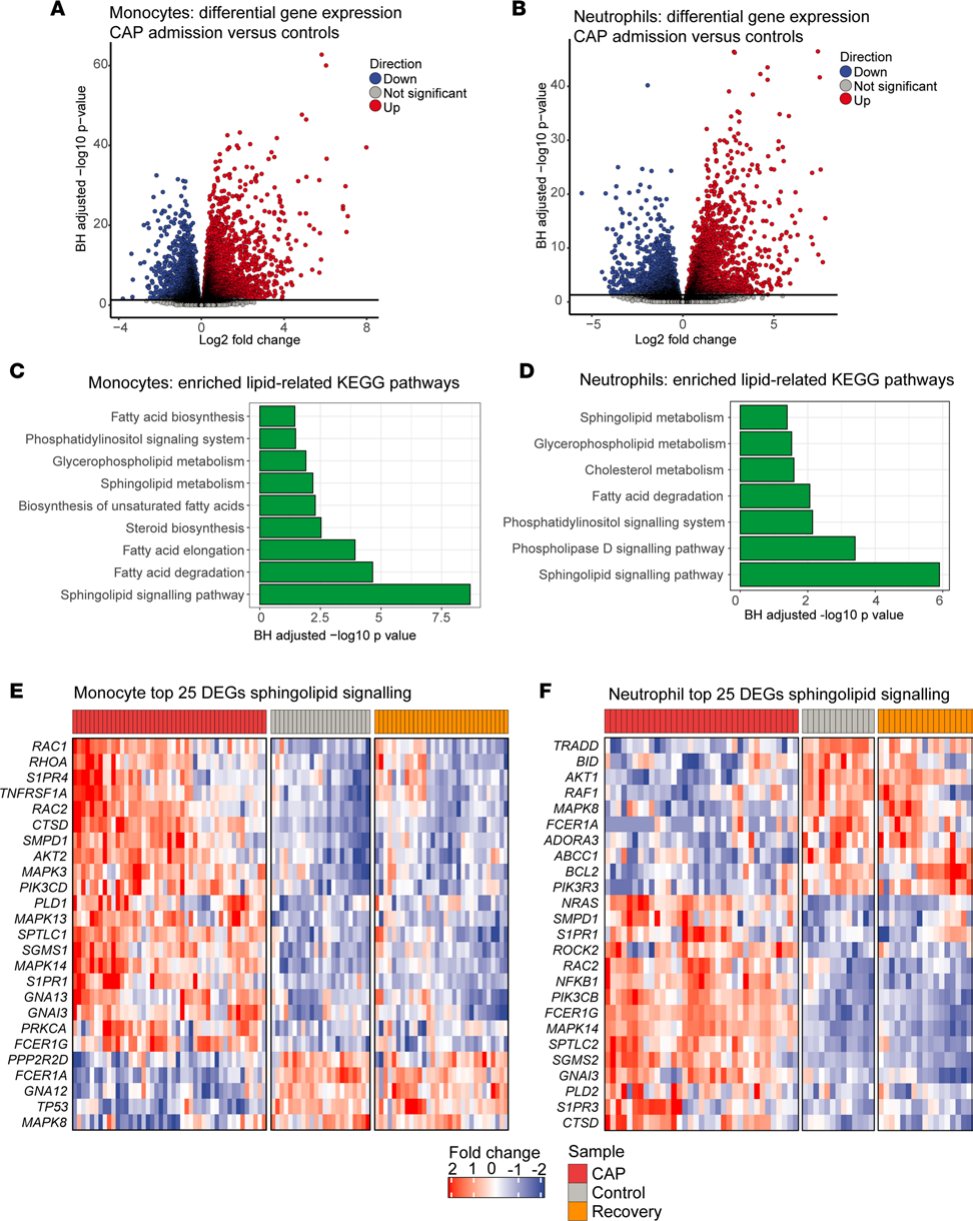
Transcriptomic analysis highlights altered sphingolipid signaling in monocytes and neutrophils. (**A** and **B**) Volcano plots showing the differentially expressed genes in monocytes and neutrophils when comparing patients with CAP to controls. Each dot represents a gene, while the color indicates whether the gene is significantly upregulated (red), significantly downregulated (blue), or not significantly altered (gray). The *x* axis denotes the log_2_(fold change) between groups, while the *y* axis shows the Benjamini-Hochberg–adjusted –log_10_(*P* value). (**C** and **D**) Bar plots showing significantly enriched lipid-related pathways based on the KEGG database in monocytes and neutrophils. The *x* axis displays the Benjamini-Hochberg–adjusted –log_10_(*P* value). (**E** and **F**) Heatmaps showing the top 25 most differentially expressed genes in the sphingolipid signaling pathway in monocytes and neutrophils, comparing patients with CAP at admission, controls, and CAP recovery samples.

Gene expression analysis identified 7126 differentially expressed genes (DEGs) between monocytes of controls and patients with CAP, and 4647 DEGs in neutrophils (Figure 4, A and B). To examine lipid-related transcriptional patterns, we performed a robust pathway analysis approach with PathfindR (22), leveraging the Kyoto Encyclopedia of Genes and Genomes (KEGG) database (23). As we aimed to integrate lipidomic with transcriptomic data, we filtered our untargeted analysis for enriched pathways categorized under “Lipid metabolism” in KEGG, as well as known lipid signaling pathways listed in this database. This approach yielded 9 significantly enriched, lipid-related pathways in monocytes, and 7 significantly enriched lipid-related pathways in neutrophils (Figure 4, C and D). Remarkably, the most significantly enriched pathway in both cell types was sphingolipid signaling; several fatty acid metabolism pathways were enriched in monocytes, while several pathways related to phospholipid metabolism were enriched in neutrophils, which together with the observed PC and PE changes may reflect extensive membrane remodeling. Most pathways showed overall upregulation during CAP, which returned to expression levels comparable to controls after CAP recovery (Supplemental Figure 5, A and B). We plotted the top 25 DEGs in the sphingolipid signaling pathway for both cell types, which also indicated an overall return to control levels after CAP recovery (Figure 4, E and F).

In conclusion, transcriptional sphingolipid signaling is significantly altered both in monocytes and neutrophils during CAP, which normalizes 1 month after hospital admission.

### Integrating lipidomic and transcriptomic data in sphingolipid signaling.

**Figure 5:**
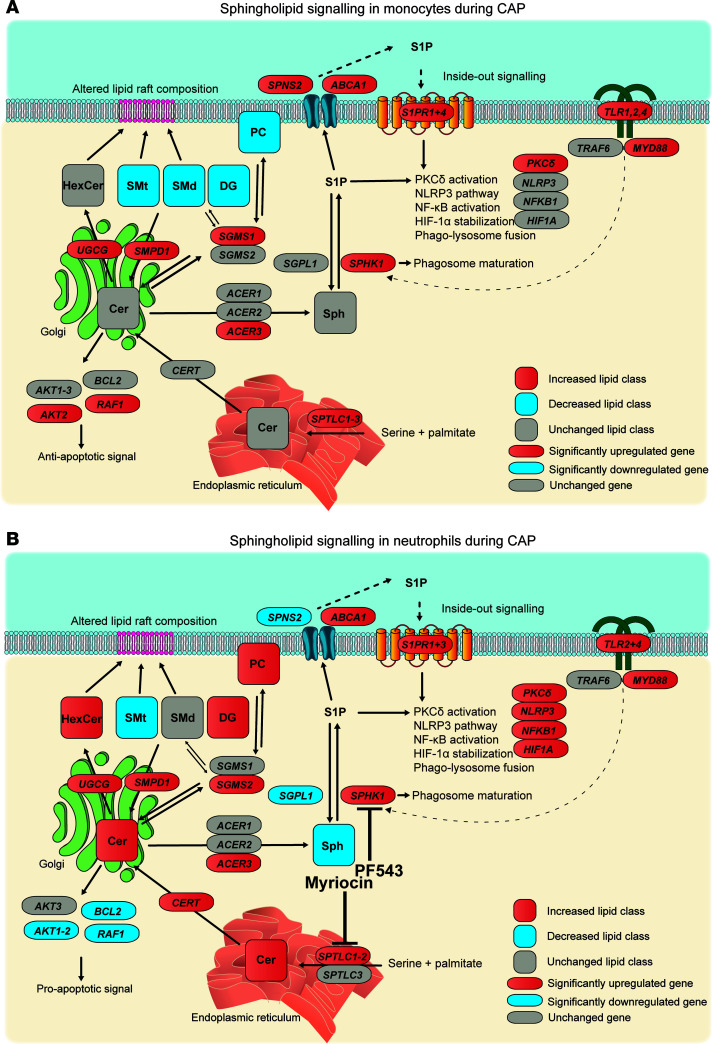
Altered sphingolipid metabolism and signaling in monocytes and neutrophils during pneumonia. (**A** and **B**) Graphical overviews of the sphingolipid metabolism and signaling pathways in monocytes and neutrophils during CAP, based on literature and the KEGG database. Ellipses represent genes, and rectangles represent the class-wide lipid levels. Red indicates significantly increased, blue indicates significantly decreased, and gray indicates nonsignificance. Gene expression analyses were corrected for multiple testing with the Benjamini-Hochberg method, in an untargeted analysis (transcriptome-wide correction).

We further focused on sphingolipid signaling, as this was the most significantly enriched lipid-related pathway in the transcriptomes of both monocytes and neutrophils, and the lipidomic analyses revealed changes in sphingolipids (SM and SPH; Figure 2) in both cell types. Moreover, bioactive sphingolipid mediators are ascribed a wide range of key leukocyte functions, including roles in cell growth and apoptosis, inflammation and immunity, cell adhesion and migration, energy metabolism, and autophagy (24). To analyze sphingolipid metabolism in more detail, we integrated the gene expression and lipidomic data based on literature and the KEGG database (Figure 5, A and B). Although the sphingolipid pathways are highly interconnected and complex, with many reversible reactions, this approach yielded several insights.

The conversion of palmitate and serine, through dihydrosphinghosine as facilitated by *SPTLC*-encoded proteins, to ceramide in the endoplasmic reticulum was upregulated in both cell types, but total ceramide levels were increased only in neutrophils. Ceramide is shuttled to the Golgi apparatus by ceramide transfer protein — encoded by *CERT*, which was only significantly upregulated in neutrophils — where it functions as a hub in the sphingolipid metabolism network. Firstly, ceramide itself can act as a potent second messenger and induce apoptosis (25). Indeed, only in neutrophils we observed a significant downregulation of *AKT1-2*, *BCL2*, and *RAF1*, which indicates a proapoptotic signature (26, 27). Secondly, ceramides can be glycosylated by ceramide glucosyltransferase, encoded by *UGCG*, to synthesize glycosphingolipids (28). *UGCG* was upregulated in both monocytes and neutrophils of patients with CAP, although this only resulted in higher hexosylceramide levels in neutrophils (Figure 5, A and B). Ceramides can also be converted into SMs — which are important parts of lipid rafts — by phosphatidylcholine:ceramide cholinephosphotransferase 1 and 2 (encoded by *SGMS1* and *SGMS2*), which requires a phosphocholine moiety from PC and also produces DG. In reverse, SMs can be converted into ceramide by acid sphingomyelinase (*SMPD1*) in the lysosome, which was upregulated in both cell types and may explain the low SM levels. Finally, ceramide can be recycled back to sphingosine by alkaline ceramidase, encoded by *ACER* genes. Sphingosine can be phosphorylated by sphingosine kinase 1 (encoded by *SPHK1*) in the cytosol, or by sphingosine kinase 2 (*SPHK2*) in the nucleus, which yields the extensively researched lipid mediator S1P. SPHK1, which can be induced by TLR2, TLR3, or TLR4 signaling (29) and is required for phagosome maturation (30), was upregulated in both cell types. We observed a clear transcriptional activation of S1P synthesis in both monocytes and neutrophils of patients with CAP, with upregulation of *SPHK1* and — only significant in neutrophils — downregulation of *SGPL1*, the gene encoding the irreversible S1P degrader S1P-lyase.

In a process called inside-out signaling, S1P must first be transferred out of the cell — either by promiscuous ABC transporters or the dedicated SPNS2 transporter — whereafter binding to the S1P G protein–coupled receptors kick-starts a plethora of downstream intracellular effects (31). *SPNS2* was significantly upregulated in monocytes and downregulated in neutrophils, while *ABCA1* was upregulated in both leukocyte subsets. Several genes for S1P receptors (*S1PR*s) were significantly upregulated in both cell types, indicating activation of S1P signaling. Downstream intracellular effects of S1P and S1PRs include HIF-1α stabilization (32, 33), NLRP3 activation (30), NF-κB activation (34), and protein kinase Cδ activation (35); these gene expression programs were particularly upregulated in neutrophils.

Taken together, expression of genes in sphingolipid metabolism showed an overall similar profile between monocytes and neutrophils during CAP, although changes in class-wide abundances of sphingolipids were most pronounced in neutrophils. This integrated analysis indicates activation of ceramide and S1P synthesis, and a decrease in SM production in immune cells during pneumonia.

### Inhibition of SPT and SPHK1 blunts cytokine production in monocytes and neutrophils.

**Figure 6:**
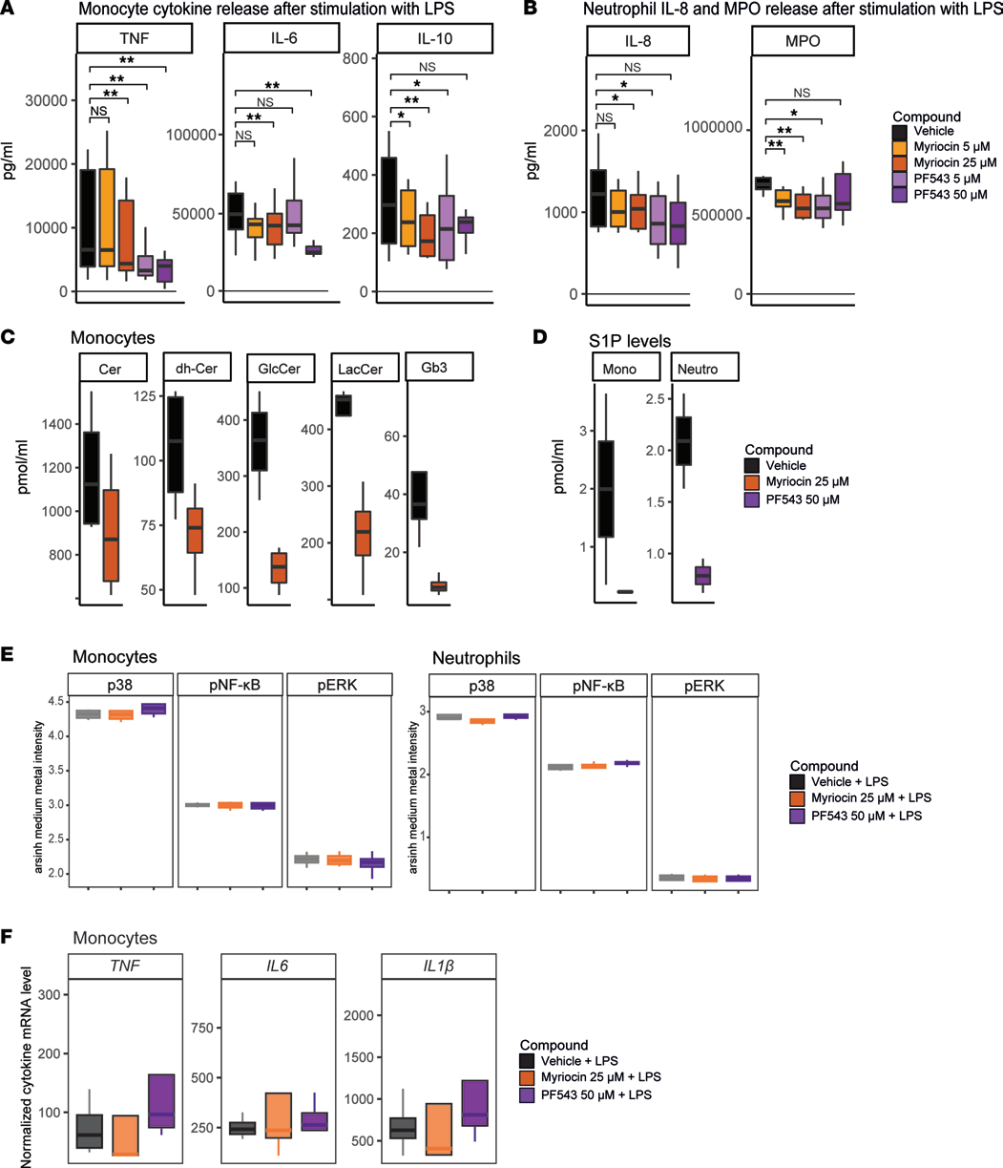
Inhibition of SPT and Sphk1 blunts LPS-induced cytokine release by monocytes and neutrophils ex vivo. (**A**) Box-and-whisker plots depicting cytokine release of monocytes (500,000 cells per well, 10 healthy donors) preincubated for 1 hour with either myriocin or PF543, and subsequently stimulated for 24 hours with lipopolysaccharide (LPS; 100 ng/mL). The *y* axis depicts concentration in pg/mL (±SEM, vertical bars). The colors indicate the different conditions. Significance was determined by a paired Wilcoxon’s rank-sum test. A *P* value below 0.05 was considered significant. *P < 0.05, **P < 0.01. (**B**) Identical to panel **A**, but here for neutrophils (500,000 cells per well, 8 healthy donors), stimulated for 2 hours with LPS. (**C**) Box-and-whisker plots showing levels (in pmol/mL) of ceramide species in monocytes incubated with either vehicle or myriocin, measured by targeted MS (*n* = 4 per condition). Cer, ceramide; dh-Cer, dihydroceramide; glcCer, glucosylceramide; lacCer, lactosylceramide; Gb3, globotriaosylceramide. (**D**) Box-and-whisker plots showing levels (in pmol/mL) of S1P in monocytes and neutrophils incubated with either vehicle or PF543, measured by targeted MS (*n* = 4 per condition). S1P, sphingosine-1-phosphate. (**E**) Box-and-whisker plots showing intensity levels of intracellular phosphorylation markers p-ERK-171Yb (pERK), p-NF-κB-Er166 (p-NF-κB), and p-p38-Gd156 (pp38) in monocytes and neutrophils after LPS stimulation and treatment with either myriocin or PF543, measured by cytometry by time of flight (CyTOF). The *y* axis indicates the arsinh-normalized medium intensity levels. There were no significant differences (*n* =4 per condition). (**F**) Box-and-whisker plots showing mRNA levels for key cytokines in monocytes treated with vehicle, myriocin, or PF543, measured by qRT-PCR. There were no significant differences (*n* = 4 per condition).

Finally, we sought to link the rewired sphingolipid metabolism to immune functionality. In this context, SPHK1 (which phosphorylates sphingosine to produce S1P; ref. 36) and SPT (serine palmitoyltransferase, which constitutes the first and rate-limiting step in the novo biosynthetic pathway of sphingolipids and ceramide) have been previously identified as regulators of inflammatory responses (37–40). Moreover, *SPTLC1* and *SPTLC2* were in the top 25 differentially expressed sphingolipid genes in monocytes and neutrophils of patients with CAP respectively, which also positioned SPT as an interesting target. We treated purified monocytes and neutrophils from noninfectious controls with myriocin (inhibiting SPT) and PF543 (a potent and highly selective inhibitor of SPHK1, leading to strongly decreased S1P and increased SPH levels, respectively; ref. 41). Cell viability was unaffected. Treatment with both myriocin and PF543 significantly reduced the capacity for cytokine release in both monocytes and neutrophils (Figure 6, A and B), in which the effect of PF543 appeared the strongest. Targeted sphingolipidomic MS confirmed that treatment with myriocin resulted in decreased levels of relevant ceramide classes (only analyzed in monocytes, Figure 6C), and that SPHK1 inhibition with PF543 decreased intracellular S1P levels in both cell types (Figure 6D). Next, we sought to understand how SPT and SPHK1 inhibition resulted in blunted cytokine release. To investigate whether altered phosphorylation of key inflammatory proteins may play a role, we measured intracellular phosphorylation markers p-ERK-171Yb (ERK), p-NF-κB-Er166 (NF-κB), and p-p38-Gd156 (p38) after LPS stimulation through cytometry by time of flight (CyTOF), and the effects of myriocin and PF543 thereon. We found that inhibiting SPHK and SPT had no effect on the phosphorylation of these inflammatory hub proteins (Figure 6E). To assess potential downstream transcriptomic effects, we incubated monocytes with LPS and the sphingolipid inhibitors, and subsequently measured the levels of key cytokine RNAs through quantitative reverse transcription PCR (qRT-PCR). We found no significant effect of the sphingolipid inhibitors on RNA levels (Figure 6F), suggesting a posttranscriptional effect.

Together, these results show that SPT and SPHK1 inhibition — and the respective decrease in ceramides and S1P — blunts the capacity for cytokine release in both monocytes and neutrophils, although the precise underlying mechanisms remain unknown. These data underline the importance of sphingolipid metabolism for immune functionality, and suggest that the observed sphingolipidomic changes in immune cells of patients with CAP may facilitate increased cytokine production during infection.

## Discussion

In this investigation, we comprehensively described the shifting lipidomic landscape of monocytes and neutrophils during pneumonia, and connected the rewiring of sphingolipid metabolism to immune cell functionality.

It is likely that the observed changes in both cell types reflect lipidomic remodeling in anticipation of antimicrobial effector functions, triggered by a state of active infection. While we zoomed in on sphingolipid metabolism and signaling, the striking lipidomic changes in other classes — such as PC, PE, BMP, TG saturation, and fatty acids — in these immune cells also warrant further investigation. For instance, the higher abundance of the membrane lipid classes in neutrophils could point to increased membrane volume and fluidity in preparation for phagocytosis. Furthermore, the increase in plasmalogens may function to protect the integrity of the cell from the oxidative stress of increased reactive oxygen species (ROS) production; plasmalogens are preferentially oxidized over double bonds in other unsaturated membrane lipids, and oxidation of plasmalogens by ROS will halt the normally propagating process of lipid peroxidation (42). The increased levels of BMP in neutrophils, which are exclusively present in late endosomes and lysosomes and help increase lysosomal activity (17), could be important for effective phagocytosis. Decreased SM levels could reorganize the composition of lipid rafts in monocytes and neutrophils, which in turn can alter cell-cell signaling. Finally, our longitudinal analysis showed a near-complete resolution of monocyte lipidomic changes after CAP recovery, while neutrophils remained partly different from controls. As circulating neutrophils have a maximum lifespan of around 1 day, these data hint at altered myelopoiesis still 1 month after hospital admission, resulting in persistent lipidomic alterations.

Our sphingolipidomic findings build on previous studies that implicated sphingolipids in proinflammatory mechanisms. Recent work showed that *Streptoccocus pneumoniae* induced *SPHK1* expression and S1P production in human macrophages, and that Sphk1 deficiency ameliorated lung injury in a mouse model of streptococcal pneumonia (37). Another study noted increased *SPHK1* expression in PBMCs from patients with sepsis, and found that inhibition of Sphk1 with PF543 improved survival in a mouse model of polymicrobial sepsis (38). Mechanistically, the authors showed that PF543 limited human macrophage inflammatory responses by repressing NLRP3 inflammasome activation. In neutrophils, S1P was found to mediate a switch from apoptosis to activation; high S1P correlated with decreased neutrophil apoptosis levels and increased NETosis activity (43). Ceramide accumulation plays a pathological role in cystic fibrosis, and is associated with chronic pulmonary inflammation (44). Intervention with myriocin decreased ceramide levels and limited inflammation both in TNF-stimulated human respiratory epithelial cells, and in a mouse model of *Pseudomonas aeruginosa* infection (45). It is notable that both our interventions in this study can lead to lower S1P levels. The effect of PF543 — inhibiting SPHK1 — is more specific and directly decreases S1P production, whereas myriocin has more widespread effects on the sphingolipidome, which may in part depend on what salvage pathways are turned on. Together, these results broaden our understanding of the interplay between sphingolipid metabolism and inflammation, and serve to highlight the functional relevance of alterations in the cellular lipidome.

This study has limitations. We were not able to assess the potential effect of different etiologies of CAP — such as viral or bacterial pneumonia — on the cellular lipidome, as standard microbiology testing, in agreement with literature (46), identified causative pathogens in only a subset of patients. On a similar note, we performed our in vitro experimental work solely with LPS as immunostimulant. Stimulation with whole bacteria and/or viruses may induce more profound or other effects, which we did not investigate. We only studied blood cells; comparison with lipidomes in cells from the airways would have been of great interest, yet difficult to achieve due to ethical restrictions in obtaining bronchoalveolar lavage fluid in this moderately ill population. While we sought to untangle how inhibiting SPHK1 and SPT might decrease the capacity for cytokine release, we were not able to pinpoint a signaling mechanism. It could be hypothesized that the decreased cytokine release after SPT and SPHK1 inhibition is not due to altered intracellular signaling, but has more to do with changes in the cellular structure and membrane, for which the importance of sphingolipids is well established (47). Finally, it is well described that monocytes and neutrophils exist in different cell states during infection (48–50). It is likely that the observed lipidomic differences between groups are driven in part by a shift in the proportion of cellular subsets among monocytes and neutrophils. Indeed, our data hint at a role for emergency granulopoiesis in the profound effect of CAP on the neutrophil lipidome. Although technically challenging (21), it would be highly valuable to perform single-cell lipidomics, ideally coupled to single-cell transcriptomics or high-resolution flow cytometry for phenotyping, in order to expose the lipidomic changes that underpin different cell states.

Our results establish a previously underappreciated role for cellular lipids in the host response to infection. Overall, these data can trigger further lipidomic investigation in other cell types, infections, and disease states. Such approaches may inform novel, lipid-driven immunomodulatory strategies, such as leveraging cellular sphingolipid metabolism to dampen neutrophil-mediated inflammation.

## Methods

### Participant details

The ELDER-BIOME study was performed in the Amsterdam UMC, located in the Academic Medical Center (AMC) and the BovenIJ hospital in the Netherlands from October 2016 to June 2017 and October 2017 to June 2018 (ClinicalTrials.gov identifier NCT02928367; refs. 51, 52). Patients older than 18 years admitted to the hospital were screened by trained research physicians. Patients were included if they were admitted with a clinical suspicion of an acute infection of the respiratory tract, defined as the presence of at least 2 diagnostic clinical criteria (new cough or sputum production, dyspnea, tachypnea, hypoxemia, abnormal lung examination, documented fever or hypothermia, leukocytosis or C-reactive protein levels >3 times above the upper limit), combined with an evident new or progressive infiltrate, consolidation, cavitation, or pleural effusion on chest x-ray or computed tomography scan. Patients with the clinical suspicion of an aspiration pneumonia, an obvious nonrespiratory source of infection, or patients who had recently been hospitalized (for >48 hours in the previous 2 weeks), or who resided in long-term care facilities, were not considered to have a working diagnosis of CAP. In addition, patients exposed to oral and/or intravenous antibiotics within 48 hours prior to hospital admission were excluded from study participation. Age- and sex-matched participants without acute infection who presented at the outpatient clinic of the Amsterdam UMC, location AMC, were included as controls.

### Method details

#### Clinical data and sample collection.

Baseline demographics, comorbidities, and other clinical data throughout follow-up were collected for all study participants. For patients with CAP, vital parameters and severity scores, such as the (quick) Sequential Organ Failure Assessment (qSOFA) score and Pneumonia Severity Index (PSI), were calculated upon hospital admission. A total of 50 mL of heparin-anticoagulated blood was collected within 24 hours of hospital admission. A second set of samples was collected 1 month after hospital admission, either at the residence of the study participant or at the outpatient clinic of the study center.

#### Cell isolation and purity.

PBMCs were obtained by density centrifugation of diluted blood (1 part blood to 1 part pyrogen-free saline) over Ficoll-Paque PLUS (GE Healthcare Life Sciences) and washed twice with cold PBS supplemented with 0.5% sterile endotoxin-free BSA (Divbio Science Europe). Monocytes were purified from PBMCs through positive selection using magnetic beads coated with anti-CD14 antibodies (Miltenyi Biotec, catalog 130-050-201). Polymorphonuclear cells were isolated from the lowest fraction of the Ficoll-Paque separated heparin tubes, and erythrocytes were lysed with Erythrocyte Lysis Buffer (Sigma-Aldrich).

Freshly isolated monocytes and neutrophils were seeded in a polypropylene 96-well plate (0.2 × 10^6^ cells per well) and washed twice with flow cytometry staining buffer (PBS containing 0.5% endotoxin-free BSA, 2 mM EDTA, and 0.1% NaN_3_ [Merck Millipore, 1687]). Cell purity was verified via flow cytometry (FACSCanto II with FACSDiva software, BD Biosciences) using CD14-APC, CD3-PE/Cy7, CD66-FITC, CD16-AF700, and CD56-Cy7 antibodies (catalog 561383, 341101, 551479, 557920, and 335826, respectively; BD Biosciences), showing cell fraction purity of 97.7% (interquartile range [IQR], 96.1–98.5) and 94.1% (IQR, 86.0–97.3) for monocytes and neutrophils, respectively. See Supplemental Figure 6 for the gating strategies. Cell fractions (0.5 × 10^6^ monocytes and 1 × 10^6^ neutrophils per aliquot) were snap-frozen until lipidomic analysis.

#### Lipidomic measurements.

Lipidomics analysis was performed as previously described (14). In a 2 mL tube, the following amounts of internal standards dissolved in 1:1 (v/v) methanol/chloroform were added to each sample: BMP(14:0)_2_ (0.2 and 2.5 nmol), ceramide [Cer(d18:1/12:0)] (0.125 nmol), ceramide [Cer(d18:1/25:0)] (0.125 nmol), DG(14:0)_2_ (0.5 nmol), glucose ceramide [GlcCer(d18:1/12:0)] (0.125 nmol), lactose ceramide [LacCer(d18:1/12:0)] (0.125 nmol), lysophosphatidic acid [LPA(14:0)] (0.1 nmol), LPC(14:0) (0.5 nmol), LPE(14:0) (0.1 nmol), lysophosphatidylglycerol [LPG(14:0)] (0.02 nmol), PA(14:0)_2_ (0.5 nmol), PC(14:0)_2_ (2 nmol), PE(14:0)_2_ (0.5 nmol), PG(14:0)2 (0.1 nmol), ceramide phosphocholines [SM(d18:1/12:0)] (2.125 nmol), SPH(d17:0) (0.125 nmol), SPH(d17:1) (0.125 nmol), and TG(14:0)_3_ (0.5 nmol). All internal standards were purchased from Avanti Polar Lipids. Methanol/chloroform (1:1 [v/v], 1.5 mL) was added before thorough mixing. The samples (0.5 × 10^6^ monocytes and 1 × 10^6^ neutrophils) were then centrifuged for 10 minutes at 20,000*g*, and the supernatants transferred to glass vials and evaporated under a stream of nitrogen at 60°C. The residue was dissolved in 150 μL of 1:1 (v/v) methanol/chloroform. Lipids were analyzed using a Thermo Scientific Ultimate 3000 binary HPLC coupled to a Q Exactive Plus Orbitrap mass spectrometer. For normal phase separation, 5 μL of each sample was injected onto a Phenomenex LUNA silica, 250 × 2 mm, 5 μm 100 Å column. Column temperature was held at 25°C. Mobile phase consisted of (A) 85:15 (v/v) methanol/water containing 0.0125% formic acid and 3.35 mmol/L ammonia and (B) 97:3 (v/v) chloroform/methanol containing 0.0125% formic acid. Using a flow rate of 0.3 mL/min, the LC gradient consisted of dwell at 10% A for 0–1 minute, ramp to 20% A at 4 minutes, ramp to 85% A at 12 minutes, ramp to 100% A at 12.1 minutes, dwell at 100% A 12.1–14 minutes, ramp to 10% A at 14.1 minutes, and dwell at 10% A for 14.1–15 minutes. For reversed-phase separation, 5 μL of each sample was injected onto a Waters HSS T3 column (150 × 2.1 mm, 1.8 μm particle size). Column temperature was held at 60°C. Mobile phase consisted of A 4:6 (v/v) methanol/water and B 1:9 (v/v) methanol/isopropanol, both containing 0.1% formic acid and 10 mmol/L ammonia. Using a flow rate of 0.4 mL/min, the LC gradient consisted of dwell at 100% A at 0 minutes, ramp to 80% A at 1 minute, ramp to 0% A at 16 minutes, dwell at 0% A for 16–20 minutes, ramp to 100% A at 20.1 minutes, and dwell at 100% A for 20.1–21 minutes. MS data were acquired using negative and positive ionization by continuous scanning over the range of *m*/*z* 150 to 2000. Data were analyzed using an in-house-developed lipidomics pipeline written in the R programming language (http://ww.r-project.org). Lipid identification was based on a combination of accurate mass, (relative) retention times, analysis of samples with known metabolic defects, and the injection of relevant standards. Lipid classes are defined in our lipidomics pipeline in terms of their generic chemical formula, where R represents the radyl group. Upon import of the lipid database in the annotation pipeline the generic chemical formula of each lipid class is expanded by replacing the R element with a range of possible radyl group lengths and double-bond numbers. The resulting expanded list of chemical formulas is then used to calculate the neutral monoisotopic mass of each species. The reported lipid abundances are semiquantitative and calculated by dividing the response of the analyte (area of the peak) by that of the corresponding internal standard multiplied by the concentration of that internal standard (arbitrary unit, AU). We also annotated a set of lipids that lacked a specific internal standard, and were normalized on one lipid species (PE). We used PE to normalize this second data set because PE is detectable all the 4 modi we used for the lipidomics analyses (NP-POS, NP-NEG, RP-POS, and RP-NEG). As these relative values are slightly less reliable, we only analyzed these data in support of the primary lipidomic set (with internal standards), and only on a class-wide level. The notation -O indicates lipids containing an alkyl-ether group, whereas -P indicates an alkenyl-ether group. When the nature of the ether species (alkyl/alkenyl) is unknown or cannot be chromatographically separated, this is indicated by the suffix (‘). As no dedicated internal standard for ether lipids are available, we used PC(14:0)_2_ and PE(14:0)_2_ to normalize the corresponding ether lipid species, as previously described (14).

#### Transcriptomics.

Directly harvested CD14^+^ monocytes and neutrophils (both 4 × 10^6^) were stabilized in RNAprotect Cell Reagent (Qiagen) and stored at –80°C until further analysis. RNA quality was assessed by bioanalysis (Agilent), with all samples having RNA integrity numbers greater than 7. Total RNA concentrations were determined by Qubit 2.0 Fluorometer (Life Technologies).

#### Ex vivo sphingolipid inhibition to assess effects on cytokine release.

Cell concentrations of freshly isolated monocytes and neutrophils (as described above) from healthy volunteers were adjusted to 5 × 10^6^ cells/mL in RPMI 1640 medium supplemented with 10% sterile fetal calf serum (HyClone), 200 mM GlutaMax (Thermo Fisher Scientific), 100 μM pyruvate (Thermo Fisher Scientific), and 50 μg/mL gentamycin (Lonza). We seeded 500,000 cells per well in a 48-well plate with a cell-repellent surface (Greiner Bio-one). Cells were incubated for 1 hour with medium, myriocin (5 or 25 μM), or PF543 (5 or 50 μM). Cells were then stimulated, still in the presence of the inhibiting compounds, with LPS (*Escherichia coli* 0111:B4 ultrapure, 100 ng/mL; Invivogen) or medium for 24 hours (monocytes) or 2 hours (neutrophils) at 37°C with 5% CO_2_ and 95% humidity. Thereafter, monocytes and neutrophils were centrifuged for 8 minutes at 1400 rpm, after which supernatants were stored at –80°C until further analysis. To asses cell viability, cells were labeled using the Fixable Viability Dye eFluor 780 (eBioscience/Thermo Fisher Scientific) as detailed in the manufacturer’s protocol, followed by flow cytometric (Cytoflex S, Beckmann Coulter) analysis of the samples.

#### Cytokine measurements.

TNF, IL-1β, IL-6, IL-8, and IL-10 levels in supernatants were measured using a Luminex multiplex assay (R&D Systems) on a BioPlex 200 (Bio-Rad), and by cytometric bead assay (R&D Systems), following the manufacturers’ instructions.

#### Ex vivo sphingolipid inhibition to assess downstream transcriptomic and phosphorylation effects.

Isolated monocytes and neutrophils were preincubated for 1 hour at 37°C with either a vehicle or one of the sphingolipid inhibitors myriocin (25 μM, Sigma-Aldrich) or PF543 (50 μM, Sigma-Aldrich). Subsequently, the samples were exposed to LPS (100 ng/mL) at 37°C in the presence of the respective inhibitor. For samples intended for CyTOF analysis (assessing phosphorylation effects), this incubation period lasted 20 minutes, after which the cells were fixed using PBS containing 1.6% paraformaldehyde (PFA). Samples processed for RNA analysis (assessing transcriptomic effects) were incubated for 3 hours, followed by the addition of cell lysis buffer RA1 for later RNA extraction (Bioke).

#### CyTOF staining and data acquisition.

Following cellular stimulation, cells were barcoded using the Maxpar Palladium Barcoding Kit (Standard Biotools) in accordance with the manufacturer’s protocol. Subsequently, the cells were subjected to 3 washes using Cell Staining Buffer (CSB) and centrifuged at 750*g* for 5 minutes. The cells were then combined into a single tube, where Fc receptors were blocked using Fc Block (BD Biosciences) and subsequently stained for extracellular markers CD66b-152Sm and CD14-Sm154 (catalog 91H033152 and 3156019B, respectively; Standard Biotools) for a duration of 30 minutes at room temperature. After another round of washing with CSB, cells were permeabilized with methanol cooled to –20°C for 20 minutes. Following this, cells were washed and stained once again for intracellular phosphorylation markers pERK-171Yb, pNfkB-Er166, and pp38-Gd156 (catalog 3171010A, 3166026D, and 3156002A, respectively; Standard Biotools). Subsequently, the cells were washed with CSB and Maxpar PBS (Standard Biotools) and stored overnight in 2 mL of a solution containing an iridium intercalator and PFA. The next day, cells were washed once with CSB and twice with Maxpar Cell Acquisition Solution + (Standard Biotools). Prior to acquisition on the CyTOF XT (Standard Biotools), the cells were filtered through a 40-μm membrane and EQ calibration beads (Standard Biotools) were utilized for bead normalization.

#### qRT-PCR.

Total RNA of the monocyte samples was isolated with NucleoSpin columns (Bioke) according to the manufacturer’s instructions. For cDNA synthesis, M-MLV Reverse Transcriptase (Promega) was used. Expression of cytokine mRNA was measured using a Roche LightCycler 480 with SensiFAST Real-time PCR kit (Bioline).

### Quantification and statistics

#### Transcriptomic data preprocessing.

Sequencing libraries were prepared by means of the KAPA RNA HyperPrep with RiboErase (Roche) as per the manufacturer’s instructions. Libraries were sequenced using the Illumina HiSeq 4000 to generate 50-bp single-end reads. The sequence read quality was assessed using FastQC methods (version 0.11.5; Babraham Institute, Babraham, Cambridgeshire, United Kingdom). Trimmomatic version 0.39 (53) was used to trim the Illumina adapters and filter low-quality reads and ambiguous nucleotide-containing sequences. Low-quality leading (3 nucleotides) and trailing (3 nucleotides) bases were removed from each read. A sliding window trimming using a window of 4 and a phred quality score threshold of 15 nucleotides was used to access the quality of the reads. After preprocessing, the remaining high-quality reads were aligned against the human Genome Reference Consortium Build 38 (GRCh38, Ensembl 84) using Hisat2 (version 2.2.0) (54) with default parameters. Count data were generated by means of the HTSeq method (55).

#### Transcriptomic analysis.

Differential expression analyses were performed using the DESeq2 package (56). Significance was calculated using Benjamini-Hochberg–adjusted *P* values. For the pathway enrichment analysis, we performed a multistep process using the pathfindR package (22). First, all significant genes with their associated *P* values were mapped onto a protein interaction network derived from the KEGG database. Then, active subnetworks, i.e., connected regions of the network that show significant changes In expression, were identified and filtered using a greedy algorithm (57). The remaining active subnetworks were then subjected to enrichment analysis by hypergeometric distribution–based tests, using the number of significantly altered genes in each active subnetwork and KEGG pathway. The resulting enriched pathways were filtered, in which pathways with Benjamini-Hochberg–adjusted *P* values greater than 0.05 were discarded. To increase robustness, this process of active subnetwork search and enrichment analyses was repeated for 10 iterations, after which the highest *P* values were used. The overall pathway score per patient (for the supplemental heatmaps) was derived by averaging the *z* score of all genes in the respective pathway per patient.

#### CyTOF data preprocessing and analysis.

The CyTOF data were acquired in the form of fcs files after performing bead normalization and debarcoding using the software package included with the CyTOF XT instrument. Subsequently, these files were analyzed using the OMIQ software package. To improve the distribution of the data, an arcsinh transformation was applied with a cofactor of 5. Clean-up gating procedures were conducted using the iridium intercalator to isolate singlet cells and cisplatin for live/dead discrimination. Monocytes were identified based on the expression of the CD14 marker, while neutrophils were identified based on the expression of CD66b.

#### qRT-PCR.

Data were analyzed using LinRegPCR software (v.2014.4; http://LinRegPCR.nl) and all results were normalized to *Hprt* expression levels.

#### Statistics.

All statistical analyses were performed using R version 4.0.4. In the box-and-whisker plots, data are represented with a median line and a box indicating the IQR. Lipid landscape plots were constructed using the geom_density_ridges gradient function within the ggplot package (where *y* is the lipid class variable). Statistical significance of class-wide differences was determined using the 2-sided Wilcoxon’s rank-sum test, with significance defined as *P* less than 0.05. All species-specific analyses were corrected for multiple testing using the Benjamini-Hochberg method, with significance defined as an adjusted *P* value of less than 0.05.

#### Study approval.

Written informed consent was obtained from all eligible participants or their legal representatives. The study protocol was approved by the local institutional review boards (METC Amsterdam UMC reference: NL57847.018.16) and conducted according to the declaration of Helsinki.

#### Data and software availability.

The monocyte transcriptomic data generated during this study are available in the Gene Expression Omnibus of the NCBI with accession number GSE160329 (https://www.ncbi.nlm.nih.gov/geo/query/acc.cgi?acc=GSE160329). All lipidomic data presented in this manuscript will be made available on MetaboLights. The data underpinning the figures in this manuscript can be found in the Supporting Data Values file. Further information and requests for resources and reagents should be directed to the corresponding author (ARS).

## Author contributions

TVDP, WJW, and BPS conceptualized the study. MVW, AVK, MPR, and FMV developed the methodology. ARS, JMB, and HPS analyzed the data. ARS, OC, BWH, XB, NAO, AK, SJ, CCAL, MJF, and FU carried out the investigation. TVDP, BPS, WJW, ADV, DRF, and FMV provided resources. ARS, MPR, and MVW curated data. ARS and TVDP wrote the original draft of the manuscript, which was reviewed and edited by ARS, TVDP, FMV, MVE, HPS, and JMB. ARS, JMB, and HPS generated figures. TVDP, WJW, ADV, and FMV supervised the study. ARS provided project administration. TVDP, BPS, and WJW acquired funding.

## Supplementary Material

Supplemental data

Supplemental table 1

Supplemental table 2

Supporting data values

## Figures and Tables

**Table 1:**
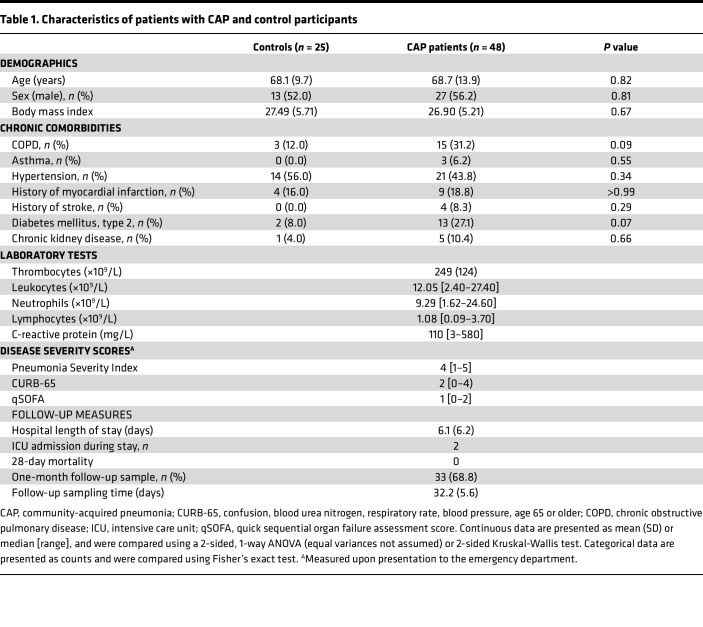
Characteristics of patients with CAP and control participants

## Version 1 02/22/2024

Electronic publication
