# Supplementary material for: The shifting lipidomic landscape of blood monocytes and neutrophils during pneumonia
Source: JCI Insight. 2024 Feb 22;9(4):e164400. doi: 10.1172/jci.insight.164400 (PMC10967382; doi:10.1172/jci.insight.164400)
Supplement: Supplemental data [file jciinsight-9-164400-s008.pdf]

Supplemental Table 2

ID	Common name	Abbr.	Synonyms	Category	Main class
BMP	Lysobisphosphatidic acid	LBPA	BMP, bis(monoacylglycero)phosphate	[GP]	Glycerophosphoglycerols [GP04]
CE	Cholesteryl ester	CE		[ST]	Sterols [ST01]
Cer	Ceramide	Cer		[SP]	Ceramides [SP02]
DG	Diacylglycerol	DG	DAG	[GL]	Diradylglycerols [GL02]
Hex2Cer[d]	Dihexosylceramide	Hex2Cer	Glc2Cer; Gal2Cer; LacCer	[SP]	Neutral glycosphingolipids [SP05]
Hex2Cer[t]	Hydroxy-dihexosylceramide	Hex2Cer		[SP]	Neutral glycosphingolipids [SP05]
HexCer[d]	Hexocylceramide	HexCer	GlcCer; GalCer	[SP]	Neutral glycosphingolipids [SP05]
HexCer[t]	Hydroxy-hexosylceramide	HexCer		[SP]	Neutral glycosphingolipids [SP05]
LPC	Lysophosphatidylcholine	LysoPC	LPC	[GP]	Glycerophosphocholines [GP01]
LPC-O	Alkyllysophosphatidylcholine	LysoPC(O)		[GP]	Glycerophosphocholines [GP01]
LPC'	Alkyl/alkenyllysophosphatidylcholine	LysoPC(')		[GP]	Glycerophosphocholines [GP01]
LPC-P	Alkenyllysophosphatidylcholine	LysoPC(P)		[GP]	Glycerophosphocholines [GP01]
LPE	Lysophosphatidylethanolamine	LysoPE	LPE	[GP]	Glycerophosphoethanolamines [GP02]
LPE-O	Alkyllysophosphatidylethanolamine	LysoPE(O)		[GP]	Glycerophosphoethanolamines [GP02]
LPE'	Alkyl/alkenyllysophosphatidylethanolamine	LysoPE(')		[GP]	Glycerophosphoethanolamines [GP02]
LPE-P	Alkenyllysophosphatidylethanolamine	LysoPE(P)		[GP]	Glycerophosphoethanolamines [GP02]
PA	Phosphatidic acid	PA		[GP]	Glycerophosphates [GP10]
PC	Phosphatidylcholine	PC		[GP]	Glycerophosphocholines [GP01]
PC-O	Alkylphosphatidylcholine	PC(O)		[GP]	Glycerophosphocholines [GP01]
PC'	Alkyl/alkenylphosphatidylcholine	PC(')		[GP]	Glycerophosphocholines [GP01]
PC-P	Alkenylphosphatidylcholine	PC(P)		[GP]	Glycerophosphocholines [GP01]
PE	Phosphatidylethanolamine	PE		[GP]	Glycerophosphoethanolamines [GP02]
PE-O	Alkylphosphatidylethanolamine	PE(O)		[GP]	Glycerophosphoethanolamines [GP02]
PE'	Alkyl/alkenylphosphatidylethanolamine	PE(')		[GP]	Glycerophosphoethanolamines [GP02]
PE-P	Alkenylphosphatidylethanolamine	PE(P)		[GP]	Glycerophosphoethanolamines [GP02]
SM[d]	Sphingomyelin/Ceramide phosphocholines	SM[d]		[SP]	Phosphosphingolipids [SP03]
SM[t]	Hydroxysphingomyelin	SM[t]		[SP]	Phosphosphingolipids [SP03]
SPH	Sphingosine, Sphinganine	SPH[d]		[SP]	Sphingoid bases [SP01]
TG	Triacylglycerols	TG	TAG	[GL]	Triradylglycerols [GL03]
TG-O	Alkyldiacylglycerol	TG-O		[GL]	Triradylglycerols [GL03]

Supplemental Table 2: Nomenclature overview of lipid classes.

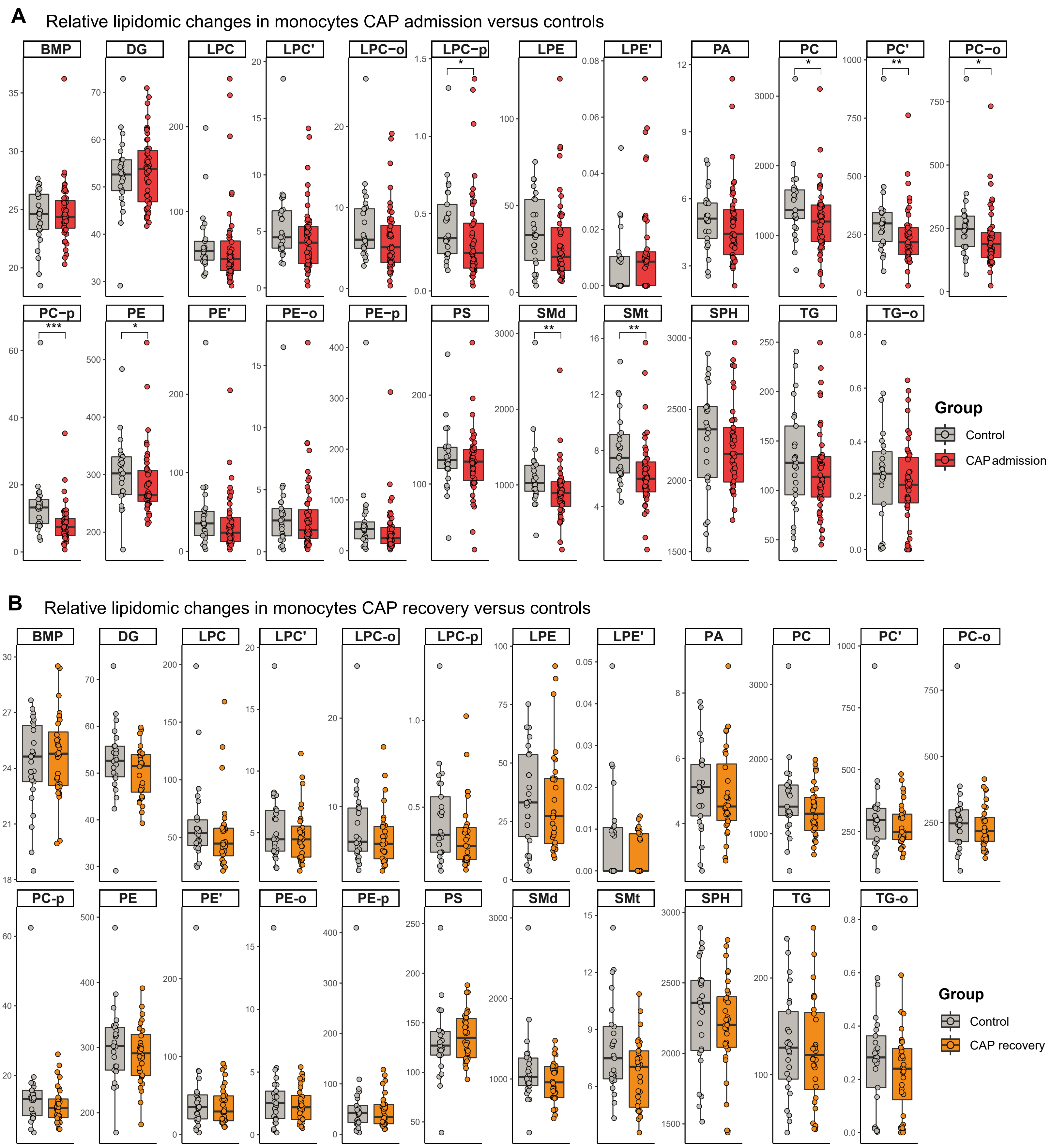

Figure S1: Relative class-wide lipidomic changes in monocytes during CAP

A) Boxplots showing class-wide lipidomic differences in monocytes between patients with CAP (N=48) and controls (N=25), as determined by a Wilcoxon-ranked sum test of aggregated values per class (a sum of all lipids per class per subject). * p-value < 0.05, ** pvalue < 0.01, *** p-value < 0.001, **** p-value < 0.0001. B) Boxplots showing classwide lipidomic differences in monocytes between patients with CAP recovery (N=33) and controls, as determined by a Wilcoxon ranked sum test of aggregated values per class (a sum of all lipids per class per subject). * pvalue < 0.05, ** p-value < 0.01, *** p-value < 0.001, **** p-value < 0.0001.

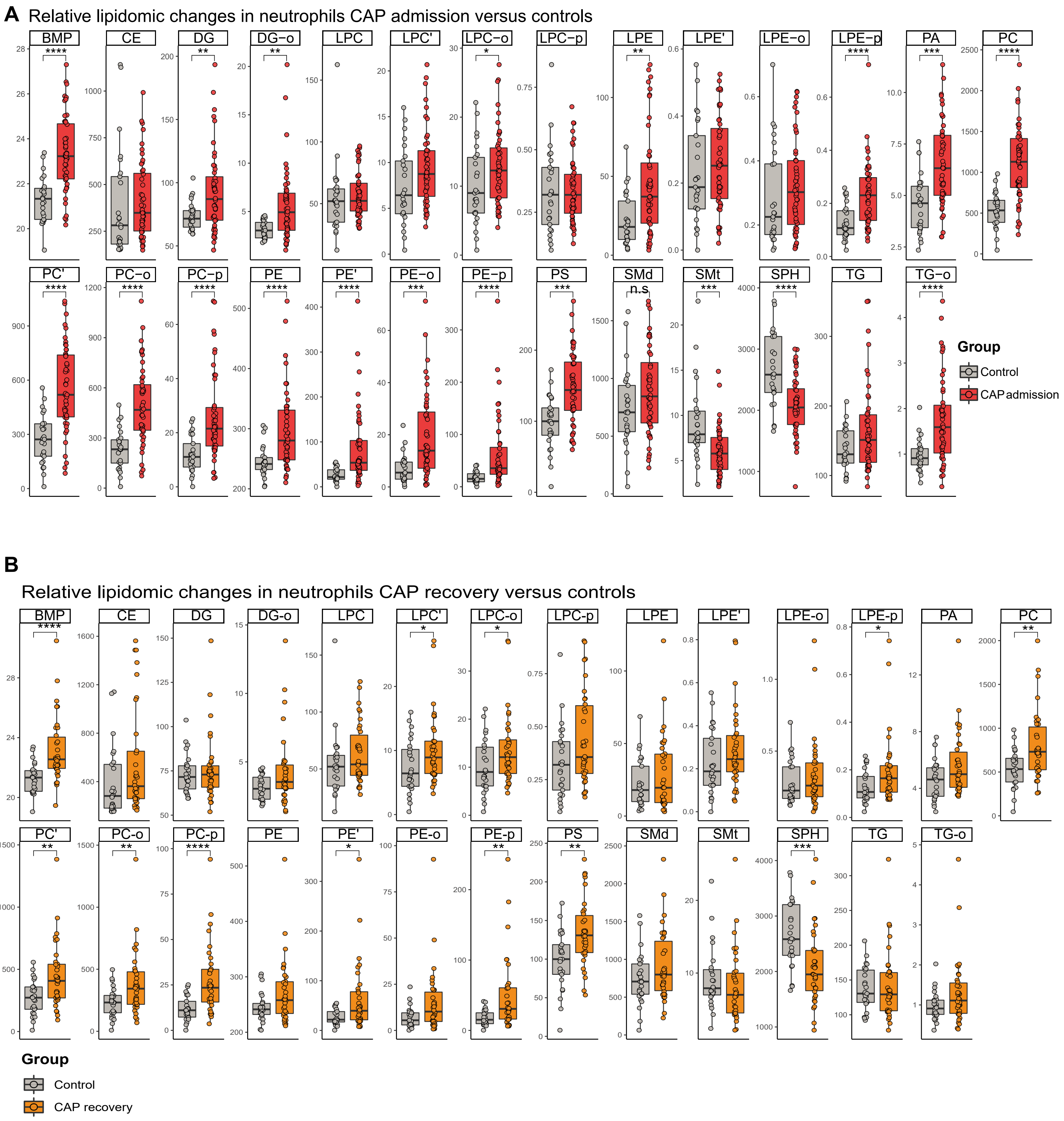

Figure S2: Relative class-wide lipidomic changes in neutrophils during CAP

A) Boxplots showing class-wide lipidomic differences in neutrophils between patients with CAP (N=48) and controls (N=25), as determined by a Wilcoxon-ranked sum test of aggregated values per class (a sum of all lipids per class per subject). * p-value < 0.05, ** pvalue < 0.01, *** p-value < 0.001, **** p-value < 0.0001. B) Boxplots showing classwide lipidomic differences in neutrophils between patients with CAP recovery (N=33) and controls, as determined by a Wilcoxon ranked sum test of aggregated values per class (a sum of all lipids per class per subject). * pvalue < 0.05, ** p-value < 0.01, *** p-value < 0.001, **** p-value < 0.0001.

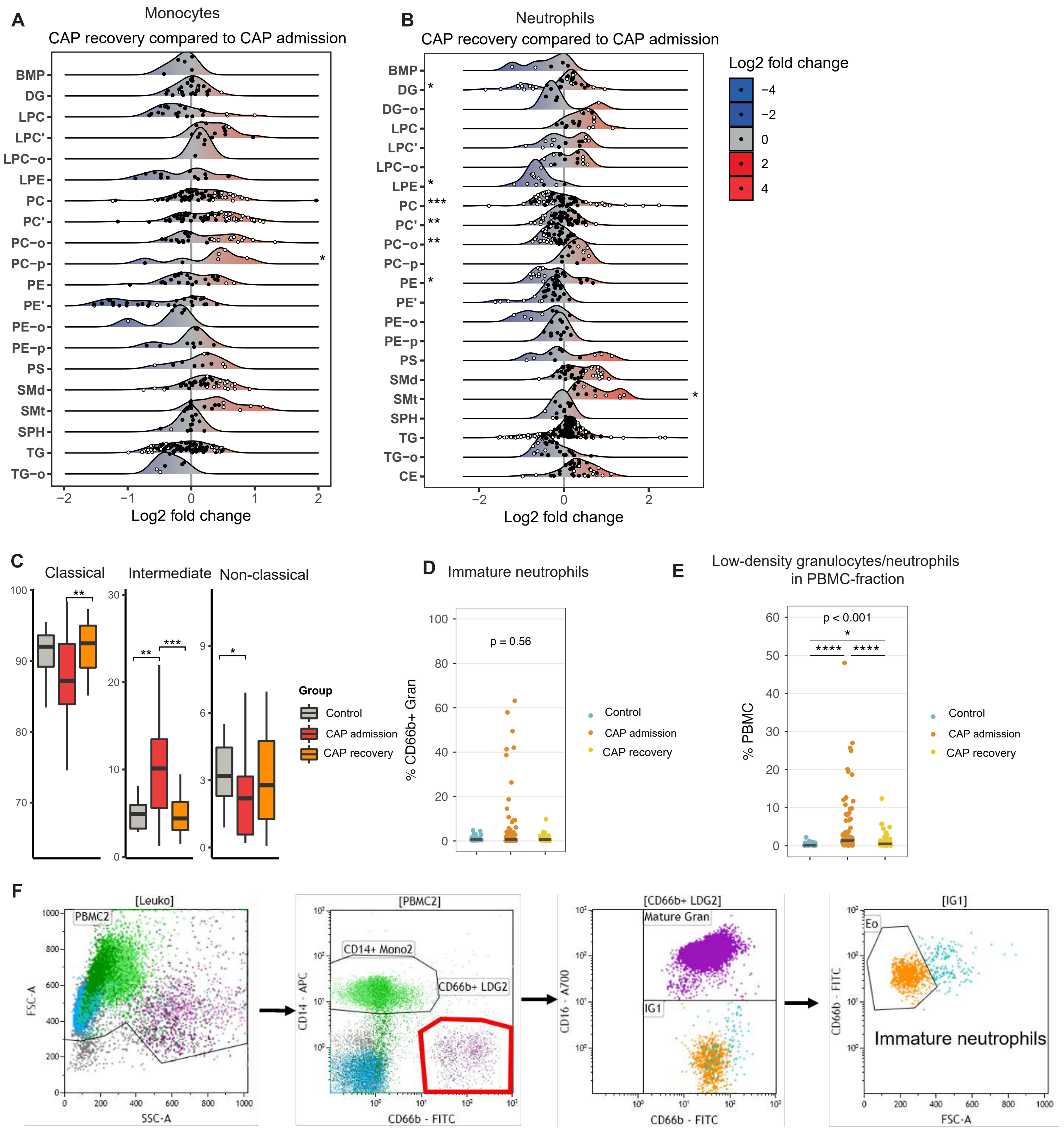

Figure S3: CAP recovery lipid landscapes and monocyte/neutrophils subsets analysis between groups

A) Monocyte lipid landscape plot, comparing CAP recovery samples to CAP admission samples in a paired analysis. Each dot represent a lipid species, which are grouped per lipid class. The color of the dots indicates whether the lipid was significantly different (white) between the groups after Benjamini-Hochberg correction for all annotated lipids. The X-axis shows the log2 fold change between groups for each lipid. The Y-axis indicates the different lipid classes, with a ridge plot per lipid class that shows the distribution of the lipids within their respective classes. On the edges of the plot, stars denote class-wide significant differences as determined by a paired Wilcoxon-ranked sum test of aggregated values per class (a sum of all lipids per class per subject). Stars on the left of the plot indicate a significant class-wide decrease, stars on the right of the plot indicate a significant class-wide increase. A p-value below 0.05 was considered significant. B) Identical to panel A, but here for neutrophils. C) Boxplot comparing the proportion of classical ($CD14^{+}CD16^{-}$), intermediate ($CD14^{+}CD16^{+}$), and non-classical ($CD14^{dim}CD16^{+}$) monocyte subsets between groups. D) Plot comparing the proportion of immature neutrophils within the neutrophil fraction between the groups. E) Plot comparing the proportion of low-density neutrophils within the polymorphonuclear cell (PBMC) fraction between groups. F) Gating strategy to identify immature low-density neutrophils within the PBMC fraction, defined as $CD14^{-}$, $CD66b^{+}$, $CD16^{low/-}$ cells. * p-value < 0.05, ** p-value < 0.01, *** p-value < 0.001, **** p-value < 0.0001.

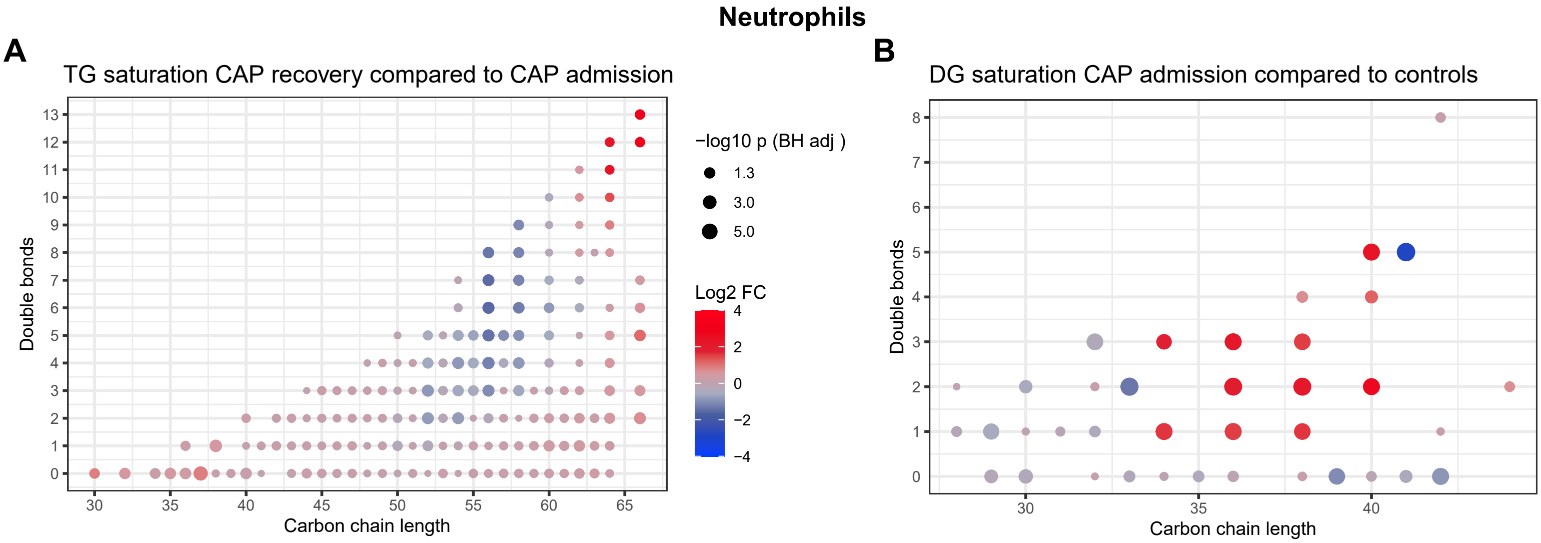

Figure S4. Saturation in neutrophil triacylglycerol and diacylglycerol
A) Dot plots of triacylglycerol saturation differences between neutrophils of CAP recovery and controls. Each dot is a triacylglycerol species. The color of the dots indicates the log2 fold change between the groups, the size of the dot is proportional to the Benjamini-Hochberg adjusted -log10 p-value of this change. The X-axis indicates the carbon chain length, the Y-axis shows the amount of double bonds. B) Identical to panel A, but here comparing diacylglycerol saturation between CAP admission and controls.

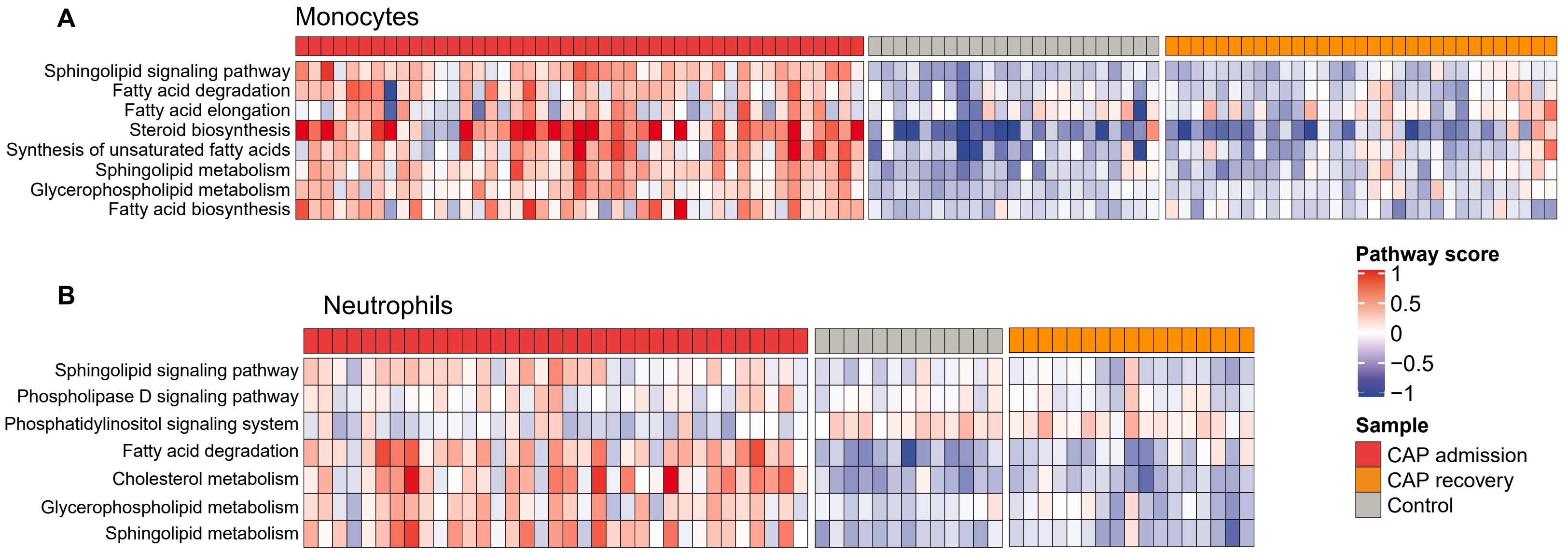

Figure S5: Mean pathway score of enriched lipid-related pathways per subject
A) Heatmap depicting the average Z-score of all genes within each enriched pathway in monocytes, per subject. The heatmap is split per group: CAP admission, CAP recovery and control samples. B) Heatmap depicting the average Z-score of all genes within each enriched pathway in neutrophils, per subject. The heatmap is split per group: CAP admission, CAP recovery and control samples.

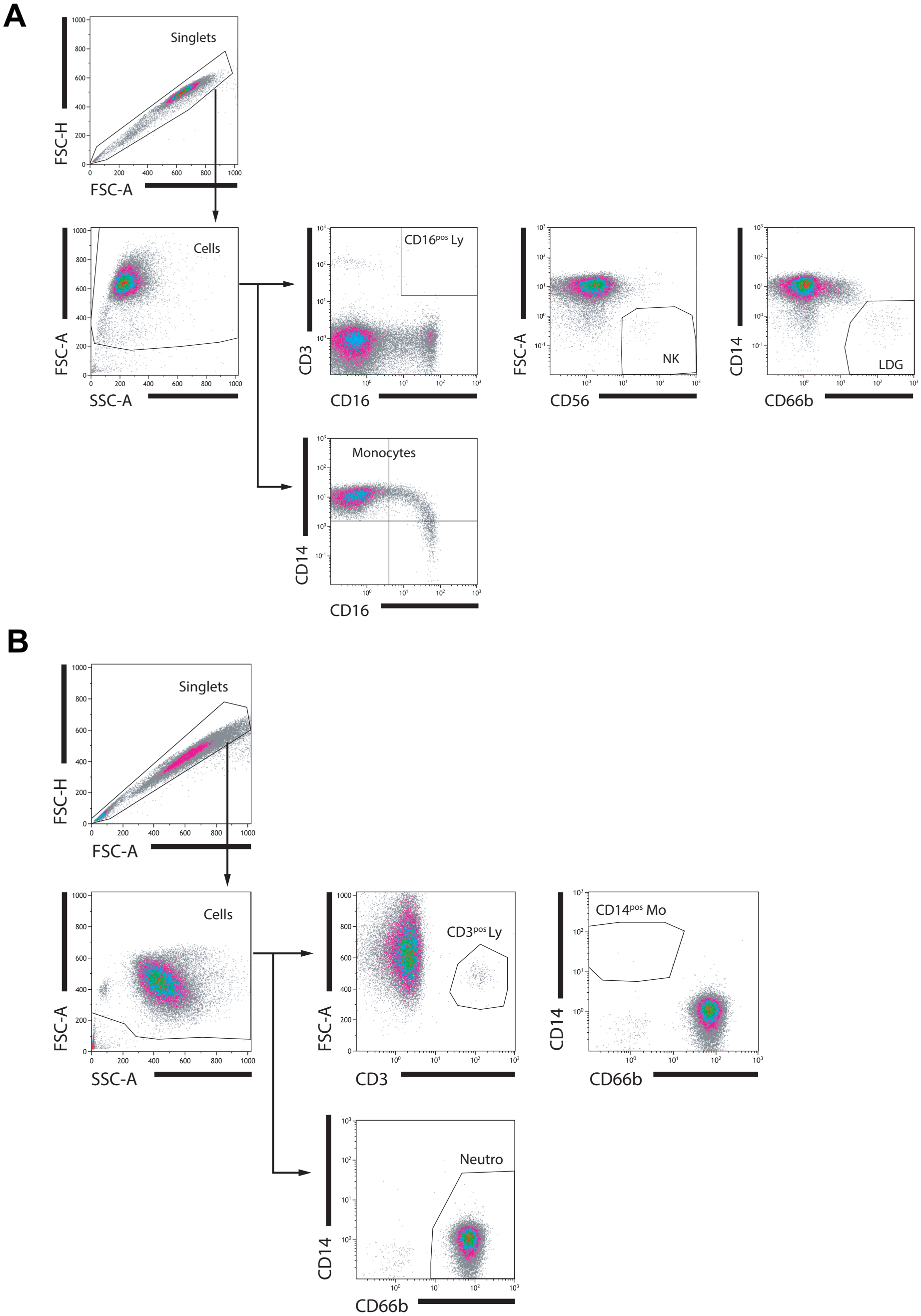

Figure S6: Gating strategy

A) Gating strategy for monocytes: all cells in the CD14/CD16 plot were identified as monocytes, consisting of classical (CD14+CD16−), intermediate (CD14+CD16+), and non-classical (CD14dimCD16+) monocyte subsets. B) Gating strategy for neutrophils.
