# Supplementary material for: The shifting lipidomic landscape of blood monocytes and neutrophils during pneumonia
Source: JCI Insight. 2024 Feb 22;9(4):e164400. doi: 10.1172/jci.insight.164400 (PMC10967382; doi:10.1172/jci.insight.164400)
Supplement: Supplemental table 2 [file jciinsight-9-164400-s010.pdf]

Supplemental Table 2

ID	Common name	Abbr.	Synonyms	Category	Main class
BMP	Lysobisphosphatidic acid	LBPA	BMP, bis(monoacylglycero)phosphate	[GP]	Glycerophosphoglycerols [GP04]
CE	Cholesteryl ester	CE		[ST]	Sterols [ST01]
Cer	Ceramide	Cer		[SP]	Ceramides [SP02]
DG	Diacylglycerol	DG	DAG	[GL]	Diradylglycerols [GL02]
Hex2Cer[d]	Dihexosylceramide	Hex2Cer	Glc2Cer; Gal2Cer; LacCer	[SP]	Neutral glycosphingolipids [SP05]
Hex2Cer[t]	Hydroxy-dihexosylceramide	Hex2Cer		[SP]	Neutral glycosphingolipids [SP05]
HexCer[d]	Hexocylceramide	HexCer	GlcCer; GalCer	[SP]	Neutral glycosphingolipids [SP05]
HexCer[t]	Hydroxy-hexosylceramide	HexCer		[SP]	Neutral glycosphingolipids [SP05]
LPC	Lysophosphatidylcholine	LysoPC	LPC	[GP]	Glycerophosphocholines [GP01]
LPC-O	Alkyllysophosphatidylcholine	LysoPC(O)		[GP]	Glycerophosphocholines [GP01]
LPC’	Alkyl/alkenyllysophosphatidylcholine	LysoPC(‘)		[GP]	Glycerophosphocholines [GP01]
LPC-P	Alkenyllysophosphatidylcholine	LysoPC(P)		[GP]	Glycerophosphocholines [GP01]
LPE	Lysophosphatidylethanolamine	LysoPE	LPE	[GP]	Glycerophosphoethanolamines [GP02]
LPE-O	Alkyllysophosphatidylethanolamine	LysoPE(O)		[GP]	Glycerophosphoethanolamines [GP02]
LPE’	Alkyl/alkenyllysophosphatidylethanolamine	LysoPE(‘)		[GP]	Glycerophosphoethanolamines [GP02]
LPE-P	Alkenyllysophosphatidylethanolamine	LysoPE(P)		[GP]	Glycerophosphoethanolamines [GP02]
PA	Phosphatidic acid	PA		[GP]	Glycerophosphates [GP10]
PC	Phosphatidylcholine	PC		[GP]	Glycerophosphocholines [GP01]
PC-O	Alkylphosphatidylcholine	PC(O)		[GP]	Glycerophosphocholines [GP01]
PC’	Alkyl/alkenylphosphatidylcholine	PC(‘)		[GP]	Glycerophosphocholines [GP01]
PC-P	Alkenylphosphatidylcholine	PC(P)		[GP]	Glycerophosphocholines [GP01]
PE	Phosphatidylethanolamine	PE		[GP]	Glycerophosphoethanolamines [GP02]
PE-O	Alkylphosphatidylethanolamine	PE(O)		[GP]	Glycerophosphoethanolamines [GP02]
PE’	Alkyl/alkenylphosphatidylethanolamine	PE(‘)		[GP]	Glycerophosphoethanolamines [GP02]
PE-P	Alkenylphosphatidylethanolamine	PE(P)		[GP]	Glycerophosphoethanolamines [GP02]
SM[d]	Sphingomyelin/Ceramide phosphocholines	SM[d]		[SP]	Phosphosphingolipids [SP03]
SM[t]	Hydroxysphingomyelin	SM[t]		[SP]	Phosphosphingolipids [SP03]
SPH	Sphingosine, Sphinganine	SPH[d]		[SP]	Sphingoid bases [SP01]
TG	Triacylglycerols	TG	TAG	[GL]	Triradylglycerols [GL03]
TG-O	Alkyldiacylglycerol	TG-O		[GL]	Triradylglycerols [GL03]
